# Neurological impairment caused by *Schistosoma mansoni* systemic infection exhibits early features of idiopathic neurodegenerative disease

**DOI:** 10.1016/j.jbc.2021.100979

**Published:** 2021-07-22

**Authors:** Juciano Gasparotto, Mario Roberto Senger, Emilio Telles de Sá Moreira, Pedro Ozorio Brum, Flávio Gabriel Carazza Kessler, Daniel Oppermann Peixoto, Alana Castro Panzenhagen, Lin Kooi Ong, Marlene Campos Soares, Patricia Alves Reis, Giuliana Viegas Schirato, Walter César Góes Valente, Bogar Omar Araújo Montoya, Floriano P. Silva, José Claudio Fonseca Moreira, Felipe Dal-Pizzol, Hugo C. Castro-Faria-Neto, Daniel Pens Gelain

**Affiliations:** 1 Departamento de Bioquímica, Centro de Estudos em Estresse Oxidativo, Instituto de Ciências Básicas da Saúde, Universidade Federal do Rio Grande do Sul, Porto Alegre, RS, Brazil; 2 Laboratório de Bioquímica Experimental e Computacional de Fármacos, Instituto Oswaldo Cruz, Fundação Oswaldo Cruz, Rio de Janeiro, RJ, Brazil; 3 Monash University Malaysia, School of Pharmacy, Bandar Sunway, Selangor, Malaysia; 4 School of Biomedical Sciences and Pharmacy and the Priority Research Centre for Stroke and Brain Injury, The University of Newcastle, Australia, Callaghan, NSW, Australia; 5 Laboratório de Imunofarmacologia, Instituto Oswaldo Cruz, Fundação Oswaldo Cruz, Rio de Janeiro, RJ, Brazil; 6 Laboratório de Fisiopatologia Experimental, Programa de Pós-Graduação em Ciências da Saúde, Universidade do Extremo Sul Catarinense, Criciúma, SC, Brazil

**Keywords:** *Schistosoma mansoni*, neuroinflammation, tauopathy, amyloid-beta (Aβ), antioxidant, neurological disease, neurodegeneration, Tau phosphorylation, schistosomiasis, 4-HNE, 4-hydroxynonenal, Aβ, amyloid-β peptide, CNS, central nervous system, CSF, cerebrospinal fluid, Def, deferoxamine, DEG, differentially expressed gene, GFAP, glial fibrillary acidic protein, NAC, *N*-acetyl-cysteine, PI, pixel intensity, PZQ, praziquantel, SOD, superoxide dismutase

## Abstract

Schistosomiasis, a neglected tropical disease caused by trematodes of the *Schistosoma* genus, affects over 250 million people around the world. This disease has been associated with learning and memory deficits in children, whereas reduced attention levels, impaired work capacity, and cognitive deficits have been observed in adults. Strongly correlated with poverty and lack of basic sanitary conditions, this chronic endemic infection is common in Africa, South America, and parts of Asia and contributes to inhibition of social development and low quality of life in affected areas. Nonetheless, studies on the mechanisms involved in the neurological impairment caused by schistosomiasis are scarce. Here, we used a murine model of infection with *Schistosoma mansoni* in which parasites do not invade the central nervous system to evaluate the consequences of systemic infection on neurologic function. We observed that systemic infection with *S. mansoni* led to astrocyte and microglia activation, expression of oxidative stress-induced transcription factor Nrf2, oxidative damage, Tau phosphorylation, and amyloid-β peptide accumulation in the prefrontal cortex of infected animals. We also found impairment in spatial learning and memory as evaluated by the Morris water maze task. Administration of anthelmintic (praziquantel) and antioxidant (*N*-acetylcysteine plus deferoxamine) treatments was effective in inhibiting most of these phenotypes, and the combination of both treatments had a synergistic effect to prevent such changes. These data demonstrate new perspectives toward the understanding of the pathology and possible therapeutic approaches to counteract long-term effects of systemic schistosomiasis on brain function.

Schistosomiasis is a parasitic disease caused by digenetic trematode flatworms of the *Schistosoma* genus. Schistosomes have a complex life cycle, using snails and mammals as intermediate and definitive hosts, respectively. Schistosomiasis is considered a neglected tropical disease, and the World Health Organization estimates that at least 250 million people are diagnosed with schistosomiasis in 78 countries and that over 779 million people live at risk of infection, with a death rate of 35,000 per year (1). Chronic infection is associated with debilitating conditions such as anemia, stunting, and reduced physical and mental capacity (1, 2). Current treatment is based on the administration of the antischistosomal drug praziquantel (PZQ), which efficiently eliminates adult worms but has a mild effect on eggs and immature forms, not preventing parasite reinfection (1, 3).

The effects of *Schistosoma* spp. infection over neurological function are studied mostly in the context of the rare neuroschistosomiasis (4, 5). However, even though it is generally accepted that regular schistosomiasis affects cognitive processes and attention levels (6, 7, 8), the neurological consequences of the more common form of the disease are largely neglected. Recently, a meta-analysis considering school-aged children from 14 countries affected by *Schistosoma mansoni*, *Schistosoma haematobium*, and *Schistosoma japonicum* concluded that schistosomiasis was significantly associated with educational, learning, and memory deficits (9). Impairment in short-term memory and slower reaction times were also observed in school children (10), whereas in adults there are reports of reduced attention levels, affected work capacity, and impaired cognition (6, 11).

In recent years, accumulating evidence has been suggesting that systemic inflammation may be a major causative factor of neuroinflammation (12). In schistosomiasis, enduring immune stimulation by the eggs in host tissues induce granuloma and fibrosis (13). As a result, chronic systemic inflammation with particular characteristics emerges, and most of the disease’s incapacitating symptoms are associated to such state. Complex neuroimmunoendocrine alterations take place in the course of the disease and are thought to influence hypothalamic functions leading to behavioral alterations (2), but the extent at which the systemic inflammation of schistosomiasis affects the function of the central nervous system (CNS) and leads to neurological pathologies is unknown.

In idiopathic neurodegenerative conditions, chronic oxidative stress and neuroinflammation are correlated to the formation of malignant aggregates of misfolded proteins, which in turn are associated with progressive loss of neuronal cells (14). Among such proteins, the most commonly studied are amyloid-β peptide (Aβ) and the stabilizing microtubule protein Tau, both involved in Alzheimer’s disease (AD), and α-synuclein, which is considered to have a prominent role in Parkinson’s disease (15). In AD, tangles containing Aβ and Tau in its hyperphosphorylated isoform are observed in frontal, parietal, and temporal cortex (16).

The present study aimed to establish if systemic infection with *S. mansoni* affects neurological integrity and function even without the presence of parasites in the CNS, which is the condition of most of schistosomiasis cases. For this purpose, we used a murine model of *S. mansoni* infection in which parasites do not invade the brain or other CNS structures but induce all other features characteristic of the disease and lead to a chronic systemic inflammation. Furthermore, we tested the effect of oral administration of the anthelmintic drug PZQ isolated or with a combination of the thiol redox compound *N*-acetyl-cysteine (NAC) and the iron chelator deferoxamine (Def) over parameters of neuroinflammation, brain oxidative stress, and neurodegeneration, as well as in spatial learning and memory. Our observations indicate that systemic infection with *S. mansoni* leads to astrocyte and microglia reactivity, oxidative stress, Tau phosphorylation, and Aβ accumulation in the prefrontal cortex of infected animals, with consequent impairment in spatial learning and memory. Combination of anthelmintic and antioxidant treatments was more effective in inhibiting such changes when compared with isolated administration of PZQ or NAC/Def. These data open new perspectives on the understanding of the pathology and possible therapeutic approaches to counteract long-term effects of schistosomiasis over the brain function.

## Results

Increased Tau phosphorylation and oxidative stress were previously observed in the brain cortex of mice infected with *S. mansoni* for 45 days (17). To shed light on this subject, we decided to investigate the biochemical, morphological, and immunological aspects of the CNS of mice infected with *S. mansoni* at different times of infection, ranging from 30 to 105 days. Somatic and parasitological parameters of control and infected mice are shown in Supporting information (Fig. S1). Compared with the control group, *S. mansoni* infected mice had a mild decrease in body weight in all infection times, excepting at 30 days post infection (Fig. S1*A*), which is probably associated with the significant increase in adult parasite burden that occurred during the period analyzed (Fig. S1*B*). Infected mice also showed an increase in liver and spleen weight (Fig. S1, *C* and *E*, respectively) as well as in hepatosomatic and spleen-somatic indexes (Fig. S1, *D* and *F*, respectively) at all experimental times. Hepatosplenomegaly is a classical clinical hallmark of schistosomiasis that occurs due to chronic inflammation caused by eggs deposition in these organs, which confirms our model of the disease.

**Figure 1:**
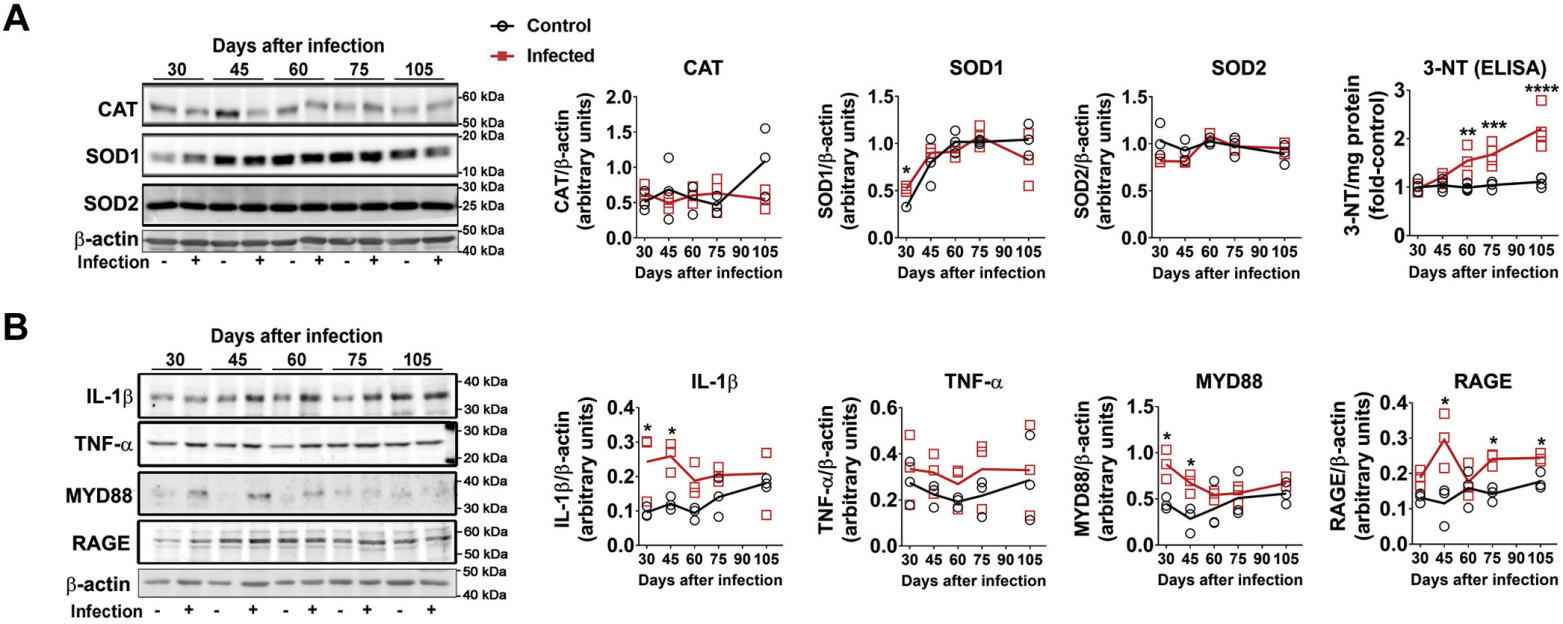
**Time-dependent effect of *S. mansoni* infection over oxidative stress and inflammation parameters in prefrontal cortex.** Five-day-old mice were infected by 150 ± 10 cercariae and euthanized at 30, 45, 60, 75, and 105 days after infection (see Fig. S1 for experimental design) to isolate prefrontal cortex tissue for Western blot (WB) and ELISA. *A*, WB quantification of antioxidant enzymes catalase (CAT), superoxide dismutase 1 (SOD1) and 2 (SOD2), and ELISA quantification of nitrotyrosine (3-NT). *B*, WB quantification of inflammatory markers IL-1β, TNF-α, MYD88, and RAGE. Representative WB gels are shown, and respective quantification graphs are depicted. Mean values ±SD are shown in graphs (n = 3 for WB, n = 4 for ELISA). Group means were evaluated by multiple *t* tests with correction for multiple comparisons with Holm-Sidak. Each *asterisk* denotes a significance degree for a minimum of *p* < 0.05.

**Figure 2:**
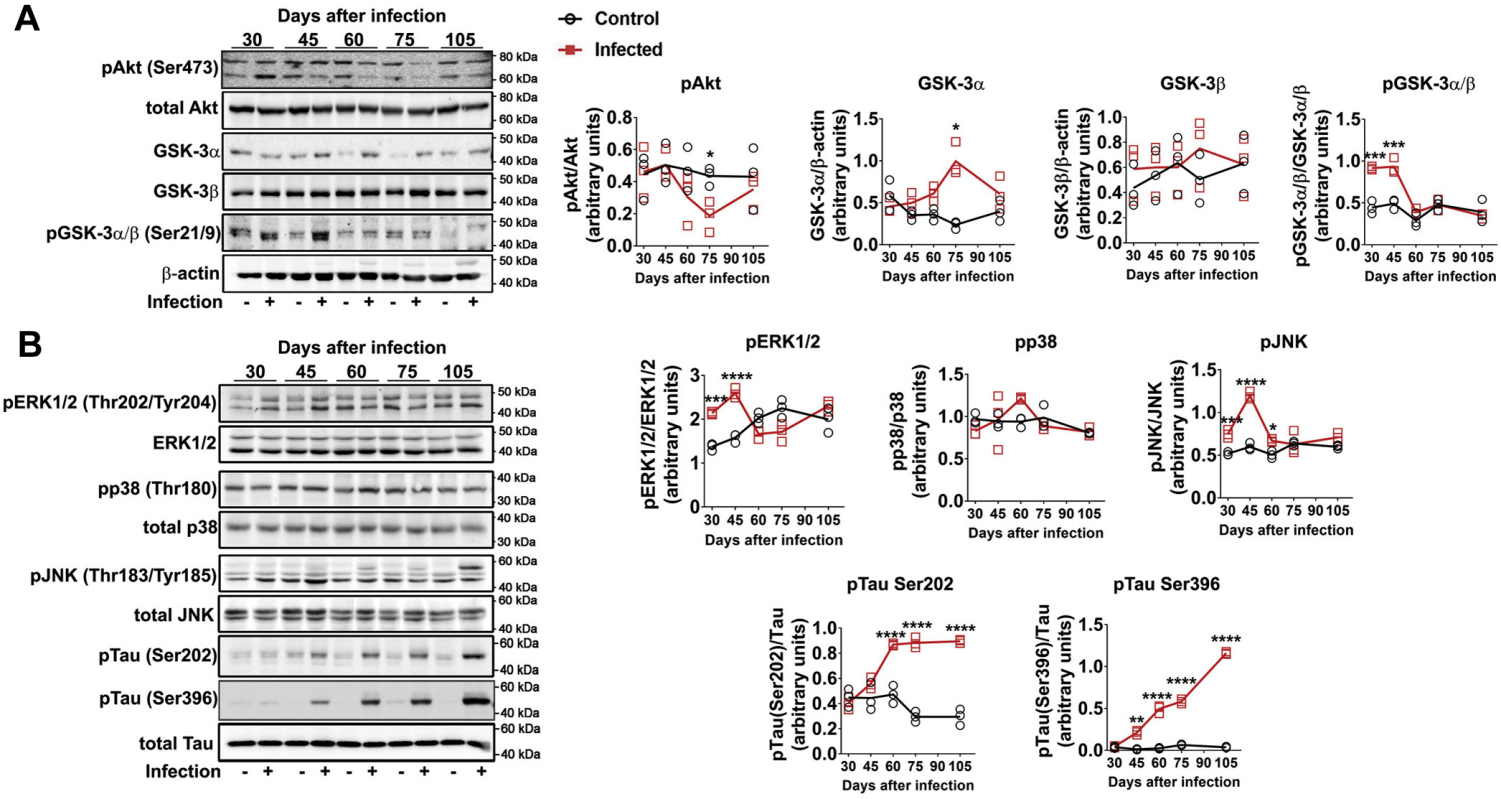
**Time-dependent effect of *S. mansoni* infection over modulation of Akt, GSK3α/β, ERK1/2, p38, JNK, and Tau phosphorylation in prefrontal cortex.** Five-day-old mice were infected by 150 ± 10 cercariae and euthanized at 30, 45, 60, 75, and 105 days after infection (see Fig. S1 for experimental design) to isolate the prefrontal cortex tissue for WB. *A*, analysis of Akt phosphorylation at Ser473, total levels of GSK3-α and GSK3-β, and relative levels of phosphorylated GSK3-α (pSer21/total) and GSK3-β (pSer9/total). *B*, analysis of the phosphorylated/total isoforms of the MAPKs ERK1/2, p38, and JNK and of the protein Tau phosphorylated at Ser202 and Ser396. Mean values ±SD are shown in graphs (n = 3 for WB). Group means were evaluated by multiple *t* tests with correction for multiple comparisons with Holm–Sidak. Each *asterisk* denotes a significance degree for a minimum of *p* < 0.05.

**Figure 3:**
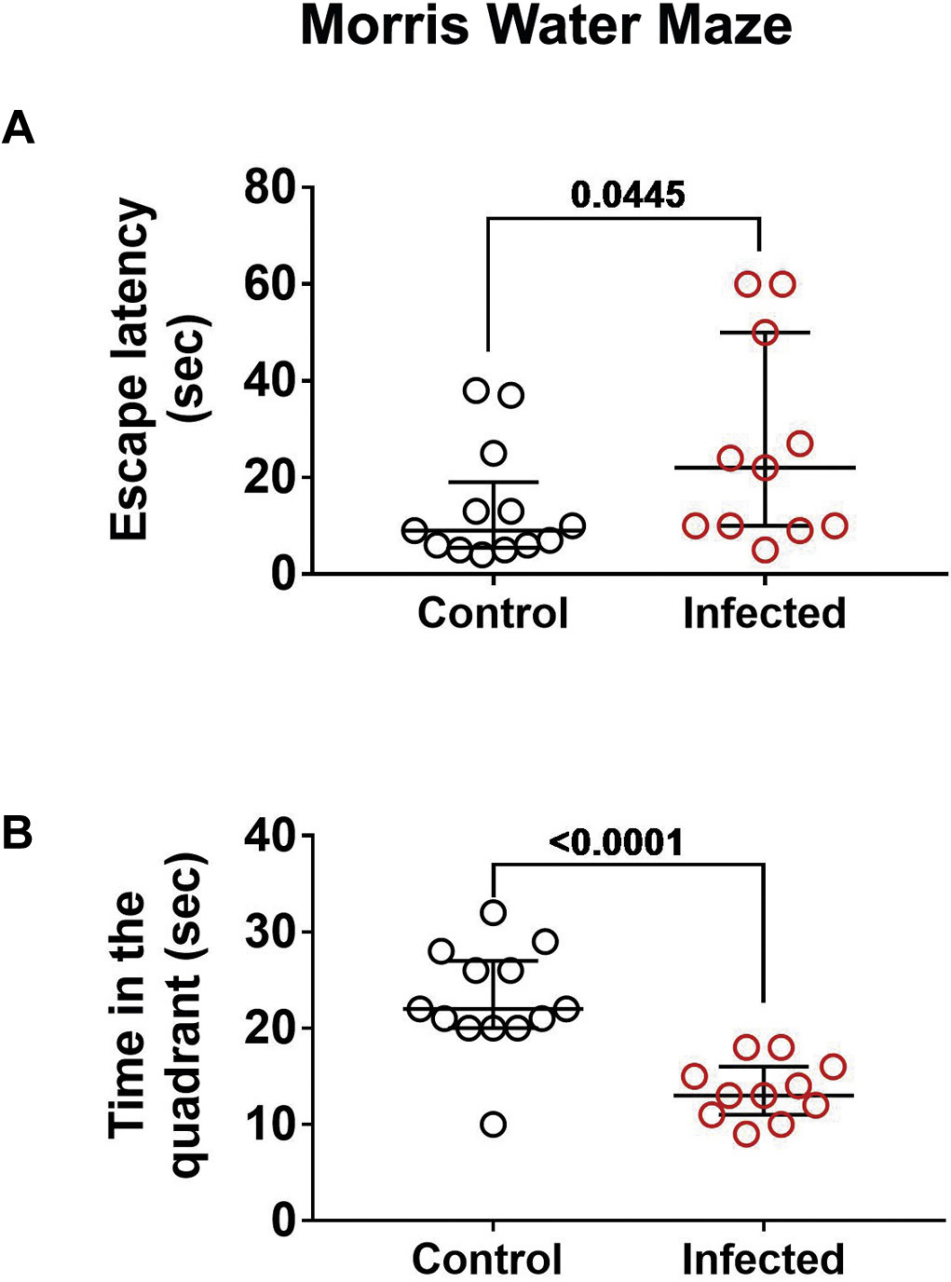
**Effect of *S. mansoni* infection over spatial memory and learning.** On the 55th day after infection, a group of mice was separated for daily training sessions at the Morris water maze task for four consecutive days. *A*, the latency time to escape to the platform was recorded at the fourth session (58th day after infection). *B*, at the fifth session (59th day after infection), the time that each animal spent in the quadrant corresponding to the subtracted platform was recorded. Median with interquartile range values are shown. Individual *p* values obtained with one-tailed Mann–Whitney test are shown.

Evaluation of oxidative stress, inflammation, and neurodegenerative markers was carried out in the prefrontal cortex of mice. The content of the antioxidant enzyme superoxide dismutase 1 (SOD1) was increased compared with control with 30 days of infection; in later periods, no differences were observed between groups, as control levels of SOD1 also increased (Fig. 1*A*). Catalase and SOD2 were not affected at any time analyzed (Fig. 1*A*). ELISA determination demonstrated that nitrotyrosine (3-NT) accumulation increased with time from 60 to 105 days after infection (Fig. 1*A*). Tissue content of TNF-α was not altered, but other cytokines or proinflammatory modulators were increased at different times of infection, namely, IL-1β, MyD88, and RAGE (Fig. 1*B*). The phosphorylation state of protein kinases commonly activated by oxidative stress or inflammation and involved in signaling pathways leading to neurodegenerative processes was evaluated next. Phosphorylation of Akt was strongly repressed at 75 days of infection, without significant changes at other time points (Fig. 2*A*). The total levels of GSK-3α and β, as well as their phosphorylated isoforms, were also assessed. GSK-3α content significantly increased at 75 days of infection, but no changes were observed in GSK-3β (Fig. 2*A*). On the other hand, the phosphorylation levels of GSK-3α/β were increased at earlier periods of infection (30 and 45 days, Fig. 2*A*). The phosphorylation levels of p38 MAPK were not changed in the brain cortex of infected animals (Fig. 2*B*). On the other hand, ERK1/2 and JNK phosphorylation were significantly increased at earlier periods (30–60 days of infection) and then returned to basal levels at later stages, with a further decrease in ERK1/2 phosphorylation compared with control at 75 days (Fig. 2*B*). As protein kinases such as ERK1/2 and GSK-3β are intimately associated with Tau hyperphosphorylation in neurodegenerative conditions (18), we also followed the time course of Tau phosphorylation (Ser202 and Ser396) and observed that phosphorylation at both sites increased with 60 days of infection, remaining elevated at 75 and 105 days (Fig. 2*B*). To evaluate if such changes were associated with neurological impairment, animals were subjected to a Morris water navigation task (Morris water maze, Fig. 3). At the 55th day after infection, animals started daily training sessions for four consecutive days, and the latency time to escape to the platform was recorded at the 58th day after infection (fourth session, Fig. 3*A*). Infected animals had increased escape latency compared with control. At the 59th day (fifth session), the time that each animal spent in the quadrant corresponding to the subtracted platform was recorded, and the infected group had a decreased average time spent in the correspondent quadrants (Fig. 3*B*). Altogether, these results suggest impairment in spatial learning and memory.

**Figure 4:**
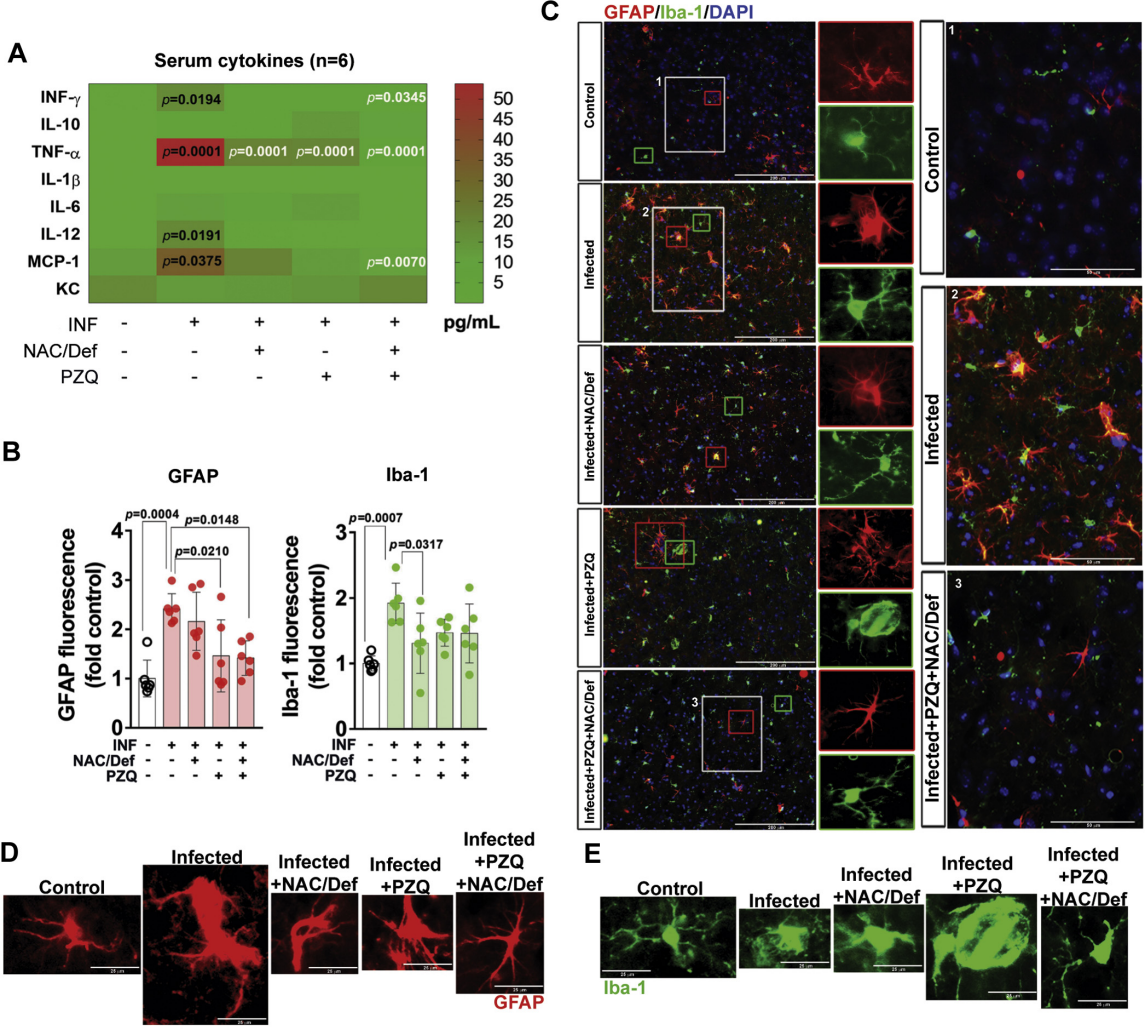
**Effect of anthelmintic and antioxidant treatments over serum inflammatory modulators, astrocytes, and microglia of mice infected with *S. mansoni*.** Five-day-old mice were infected with *S. mansoni* cercariae. Praziquantel (PZQ, 100 mg/Kg) and/or a combination of *N*-acetyl-L-cysteine and deferoxamine (NAC/Def, 200 mg/Kg) was orally administered once a day from the 42nd to the 46th day after infection. Animals were euthanized at the 60th day after infection for analysis of the serum and prefrontal cortex. *A*, serum cytokines panel was obtained with Multiplex-based immunoarray. Mean values (pg/ml, n = 6) were compared by two-way ANOVA followed by Tukey’s post hoc test with *p* values embedded in the figure (numbers in *black* denote difference respective to control group and numbers in *white* denote difference respective to infected group). *B*, quantification values (mean ± SD, n = 6) and *C*, representative images of immunofluorescence-based detection of GFAP and Iba-1 in histological sections of the prefrontal cortex. DAPI was used for nuclear staining. Scale bars in main figures correspond to 200 μm; in zoom magnification panels, scale bars correspond to 50 μm. Quantification was analyzed by two-way ANOVA followed by Tukey’s post hoc test; *p* < 0.05 values between groups are depicted. Single-cell level visualization of (*D*) GFAP and (*E*) Iba-1 immunofluorescence is shown in increased magnification, with individual images at the same scale of size. Scale bars correspond to 25 μm. Image panels in *C*–*E* are derived from the same micrographs shown in Figs. S4 and S5.

To further understand the relationship between the inflammatory and redox changes induced by *S. mansoni* infection and the neurochemical and behavioral changes observed here, we subjected infected mice to a protocol of administration of the anthelmintic PZQ (100 mg/kg) and a combination of the antioxidant *N*-acetyl-cysteine and the iron chelator deferoxamine (NAC/Def 200 mg/kg). The drugs were chosen as PZQ is the drug of choice for schistosomiasis treatment, whereas the NAC/Def combination was previously demonstrated to be neuroprotective in other models of systemic inflammation (19). The protocol of drug administration is graphically detailed in Supporting information (Fig. S2) and the animals were analyzed at the 59th (behavioral tests) and 60th (tissue collection) days of infection. Analysis of liver and spleen weight, as well as number of worms in the mesentery, confirmed that PZQ treatment was effective in eliminating worms (Fig. S2*B*). A cytokine panel array of the serum of mice with 60 days of infection demonstrated that *S. mansoni* increased the circulating levels of IFN-γ, TNF-α, IL-12, and MCP-1 (Fig. 4*A*). Isolated PZQ or NAC/Def were able to decrease TNF-α levels, whereas their combination (PZQ + NAC/Def) was efficient to decrease serum IFN-γ, TNF-α, and MCP-1. Therapeutic treatments were not capable of reducing IL-12 levels. Of interest, although circulating TNF-α was increased, IL-1β levels were not altered, as opposed to the results observed in the prefrontal cortex (Fig. 1*B*). To assess neuroinflammation status, immunofluorescence histology of the prefrontal cortex was assessed using GFAP and Iba-1 antibodies (Fig. 4, *B* and *C*). The prefrontal cortex of infected animals presented increased immunostaining for GFAP and Iba-1 (Fig. 4*B*) as well as morphological alterations in both GFAP- and Iba-1-positive cells that are indicative of astrocyte and microglia reactivity (Fig. 4, *C*–*E* and Figs. S4 and S5). GFAP immunostaining levels were not significantly changed by NAC/Def, but PZQ alone or in combination with NAC/Def inhibited the increase in GFAP immunofluorescence levels (Fig. 4*B*). Morphological alterations of GFAP-positive cells were evident among groups; astrocytes of infected animals presented swollen cell bodies and projections compatible with reactive state, and this morphological pattern was less pronounced in all other groups (Fig. 4, *C* and *D* and Fig. S4). In infected animals, it is possible to observe Iba-1-positive cells with swollen cell bodies and thicker cell projections (Fig. 4, *C* and *E* and Fig. S5). Iba-1 immunofluorescence levels were inhibited by antioxidant treatment, but PZQ did not have a significant effect; interestingly, the combination of PZQ with NAC/Def was not effective in inhibiting the increase in Iba-1 levels (Fig. 4*C*).

**Figure 5:**
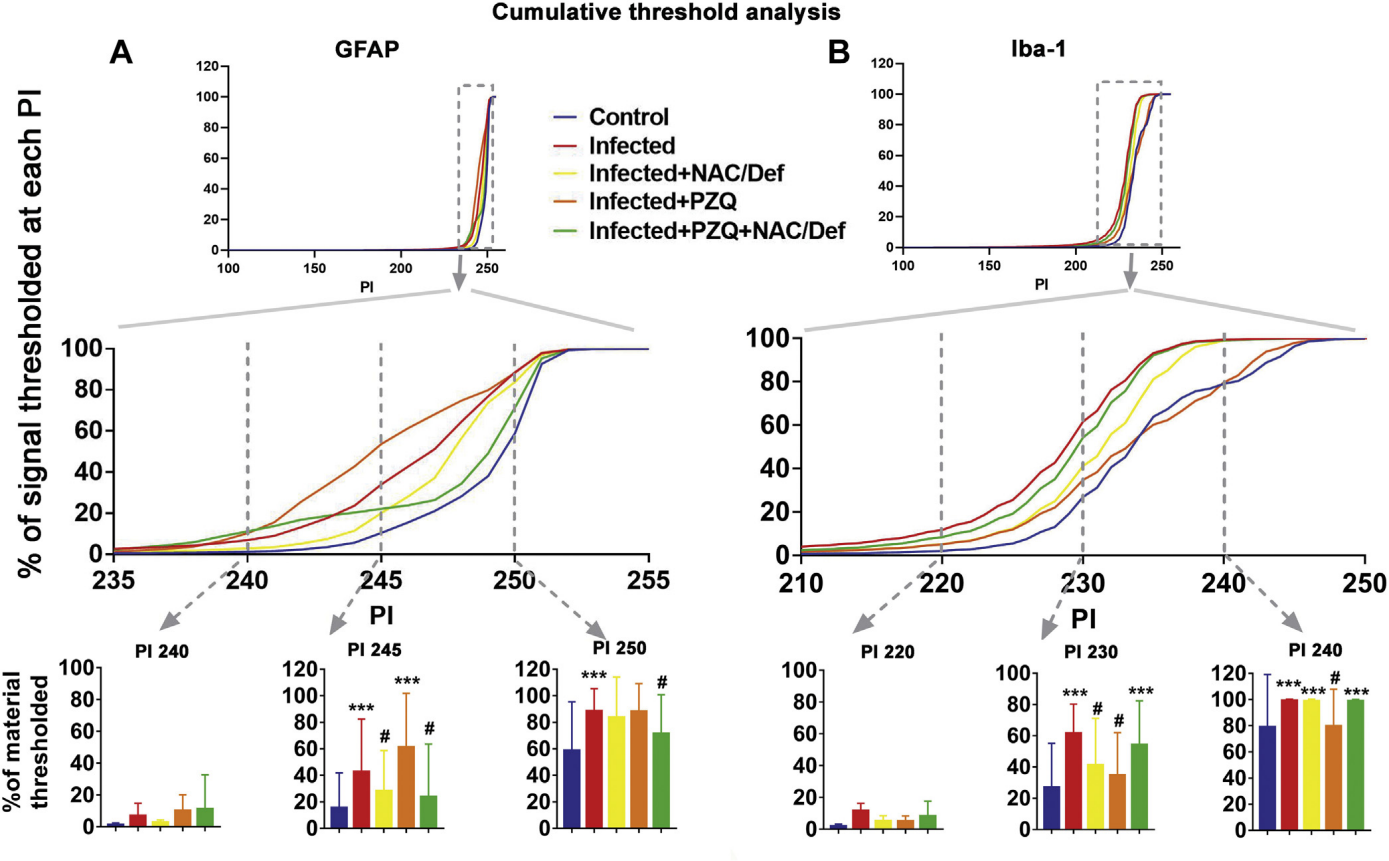
**Cumulative threshold analysis of GFAP and Iba-1 labeling in prefrontal cortex of mice infected with *S. mansoni* and treated with PZQ and NAC/Def.** Percentage of material thresholded of astrocytes (*A*, GFAP) and microglia (*B*, Iba-1) at different levels of pixel intensity (PI). At the darkest intensity spectra (PI 0–200), no differences are observed, but at lighter intensity spectra (PI 210–255), where cellular processes are mainly thresholded, differences are observed. *Bar graphs* depict the material thresholded at PIs 240, 245, and 250 for GFAP and at PIs 220, 230, and 240 for Iba-1. Intermediary PIs (245 for GFAP and 230 for Iba-1) are the most representative for statistical analysis of thresholded materials. Data were analyzed by two-way ANOVA followed by Tukey’s post hoc test, and *p* < 0.05 values between groups are depicted; *asterisks* denote significance degree of difference (for a minimum of ∗*p* < 0.05) compared with control group; #different from infected group (*p* < 0.05).

Further analysis of GFAP- and Iba-1-immunostained cells was performed by digital reconstruction to assess diverse parameters associated with cell reactivity/activation (Table 1). Infection with *S. mansoni* increased the number of GFAP- and Iba-1-positive cells, whereas soma eccentricity and cell extent were altered only in GFAP-positive cells. Treatment with PZQ and/or NAC/Def had variable effects over these parameters, either inhibiting changes or not presenting any effect (Table 1). Cumulative threshold analysis of pixel intensity in GFAP and Iba-1 immunostaining was performed, and the spectra of pixel intensity was split for quantitative analysis at specific intensities associated with cell processes (Fig. 5). In both GFAP- and Iba-1-positive cells, the infected group underwent a left shift of the thresholded signal spectra in relation to the control group. In GFAP-positive cells, quantification of the number of pixels at pixel intensity (PI) 245 demonstrated a significant increase in the infected group that was inhibited by NAC/Def alone or in combination with PZQ (Fig. 5*A*). At PI 250, only PZQ + NAC/Def had an inhibitory effect and, altogether, these data suggest that the combination of PZQ + NAC/Def has the most protective effect over astrocytes. In Iba-1-positive cells, only isolated treatments with either NAC/Def or PZQ had an inhibitory effect over the number of pixels in infected animals at PI 230, whereas at PI 240 PZQ alone is the only treatment capable of inhibiting this increase (Fig. 5*B*). These data suggest that, although PZQ alone did not affect the total levels of Iba-1 fluorescence (Fig. 4*B*), it was the more effective treatment in inhibiting microglia activation.

**Table 1:** Morphological alterations in GFAP- and Iba-1-positive cells that are indicative of astrocyte and microglia reactivity, assessed by digital reconstruction and analysis

Measure	Marker	Control (n = 4)	Infected (n = 5)	Inf + NAC (n = 4)	Inf + PZQ (n = 5)	Inf + NAC + PZQ (n = 5)	One-way ANOVA
		Mean (SD)					*p*-Value
Cell number	GFAP	4.5 (4.359)	19.4∗∗ (7.503)	11.75 (2.5)	10.2 (6.458)	9.2 (5.495)	**0.015∗**
	Iba1	31.75 (7.136)	54.6∗∗∗ (10.95)	26.75^###^ (4.349)	32.4^####^ (5.683)	28.4^####^ (3.647)	**<0.001∗**
Primary branch	GFAP	4.108 (2.6)	3.893 (0.321)	4.838 (0.906)	3.045 (1.159)	3.831 (1.095)	0.435
	Iba1	6.527 (0.641)	6.942 (1.277)	7.07 (1.295)	6.84 (0.502)	7.333 (0.786)	0.782
Total number branch	GFAP	56.81 (49.8)	10.51∗∗ (10.16)	14.65 (2.973)	15.59 (11.48)	13 (8.673)	**0.036∗**
	Iba1	12.68 (3.708)	11.32 (0.68)	14.11 (4.134)	11.64 (1.373)	16.69 (4.637)	0.101
Total branch length	GFAP	970.9 (816.1)	283.0 (232.9)	381.1 (76.04)	332.4 (204)	342.2 (202)	0.088
	Iba1	361.3 (80.75)	325.8 (20.65)	390.9 (93.98)	330.8 (24.45)	442.4 (105.7)	0.114
Cell radius	GFAP	46.05 (23.03)	28.16 (6.829)	34.25 (4.607)	30.64 (10.19)	28.51 (7.014)	0.191
	Iba1	35.26 (1.572)	35.38 (1.293)	35.86 (2.779)	34.9 (0.666)	37.32 (1.697)	0.227
Soma area	GFAP	20.48 (16.95)	17.59 (11.73)	16.86 (6.932)	17.63 (9.553)	23.73 (11.69)	0.884
	Iba1	100.1 (19.26)	111.2 (29.72)	91.47 (22.2)	108.3 (15.93)	102 (22.11)	0.726
Soma eccentricity	GFAP	0.691 (0.15)	0.879∗ (0.045)	0.885 (0.038)	0.730 (0.089)	0.841 (0.094)	**0.013∗**
	Iba1	0.814 (0.035)	0.806 (0.042)	0.799 (0.02)	0.806 (0.017)	0.809 (0.02)	0.967

Bold values indicate significant values detected by one-way ANOVA. ∗∗∗ For *p* < 0.001, ∗∗ for *p* < 0.01, and ∗ for *p* < 0.05 compared with the control group; #### for *p* < 0.0001, ### for *p* < 0.001, ## for *p* < 0.01, and # for *p* < 0.05 compared with the infected group (Tukey post hoc analysis).

**Figure 6:**
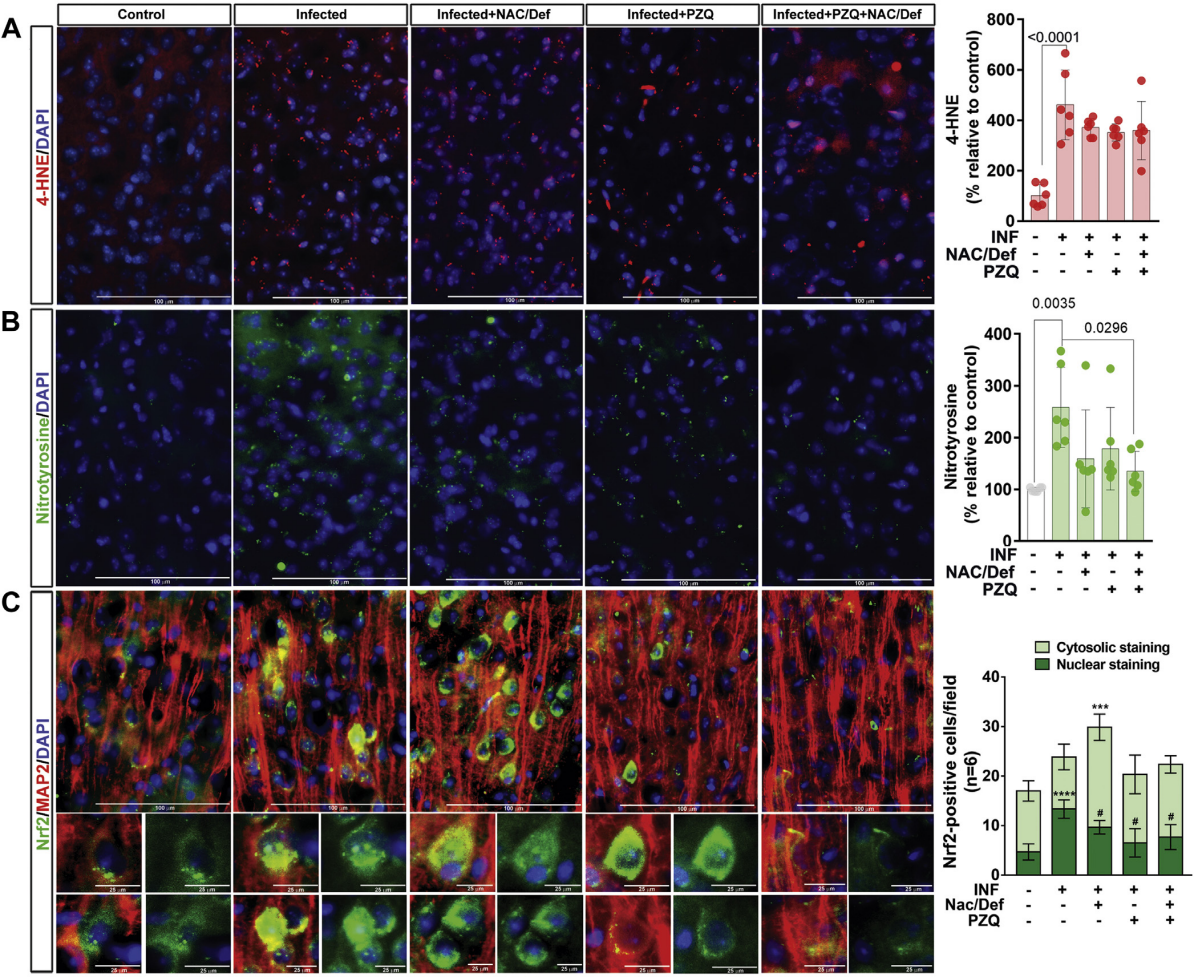
**Effect of anthelmintic and antioxidant treatments over content of 4-hydroxynonenal and nitrotyrosine and Nrf2 cellular compartmentalization in prefrontal cortex of mice infected with *S. mansoni*.** Infected mice received daily PZQ (100 mg/Kg) and/or combination of NAC/Def (200 mg/Kg) from the 42nd to the 46th days after infection and were euthanized at the 60th day after infection for isolation of the prefrontal cortex. Representative images and quantification levels (mean ± SD, n = 6) of immunofluorescence-based detection of (*A*) 4-hydroxynonenal (4-HNE) and (*B*) nitrotyrosine. *C*, immunofluorescence-based detection of Nrf2 (*green*) and the neuronal marker MAP2 (*red*). The number of cells with Nrf2 staining in either cytosol or nucleus per field was counted. DAPI was used for nuclear staining. Scale bars correspond to 100 μm in (*A*–*C*) multicell panels; in zoom magnification panels showing nuclear/cytosolic staining at (*C*), scale bars correspond to 25 μm. Data were analyzed by two-way ANOVA followed by Tukey’s post hoc test and *p* < 0.05 values between groups are depicted; *asterisks* denote significance degree of difference (for a minimum of ∗*p* < 0.05) compared with control group; #different from infected group (*p* < 0.05).

To evaluate the efficiency of anthelmintic and antioxidant treatment against brain oxidative stress, immunofluorescence detection of 4-hydroxynonenal (4-HNE) and nitrotyrosine levels in the prefrontal cortex was performed. Infected animals presented a significant increase in 4-HNE levels compared with control; no significant change was observed in animals treated with PZQ and/or NAC/Def (Fig. 6*A*). Nitrotyrosine accumulation also was enhanced in infected animals, and the combination PZQ + NAC/Def was able to decrease nitrotyrosine levels (Fig. 6*B*). Although a decrease in average values was observed with other treatments, this effect was not significant. We also analyzed Nrf2 immunostaining in prefrontal cortex samples. This transcription factor is activated by the oxidation of sulfhydryl groups in its adaptor protein Keap1, which, in turn, releases Nrf2 from its ubiquitination cycle and allows it to bind to antioxidant response elements, thus activating antioxidant genes transcription. Infected animals had increased Nrf2 staining colocalized with DAPI-stained areas (Fig. 6*C*). Administration of NAC/Def reduced nuclear Nrf2 and increased Nrf2 staining in cytosolic areas, while it also increased the total number of Nrf2-positive cells per field (Fig. 6*C*). Treatment with PZQ or PZQ + NAC/Def significantly decreased the nuclear localization of Nrf2 staining (Fig. 6*C*); these results indicate that NAC/Def administration stimulates the activation of antioxidant defense. However, considering the data in Figure 6, *A* and *B*, these results, altogether, are indicative that infected animals had increased oxidative stress and reactive species production in the prefrontal cortex and that the combination of anthelmintic and antioxidant treatment, although not effective in decreasing 4-HNE accumulation, still is the optimal approach to prevent most of the effects of oxidative stress.

**Figure 7:**
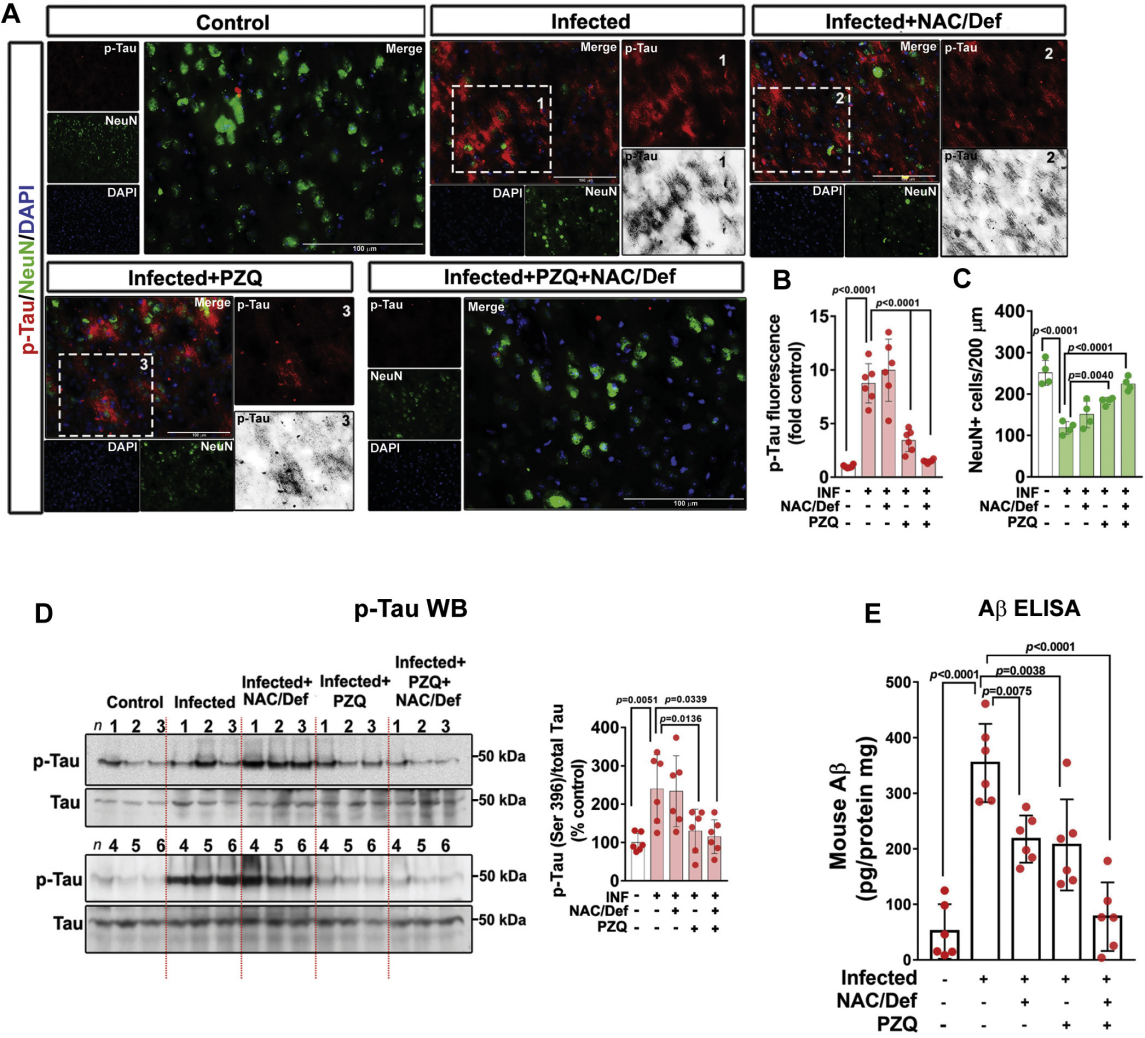
**Effect of anthelmintic and antioxidant treatments over Tau phosphorylation and β-amyloid (Aβ) accumulation in prefrontal cortex of mice infected with *S. mansoni*.** Infected mice received daily PZQ (100 mg/Kg) and/or combination of NAC/Def (200 mg/Kg) from the 42nd to the 46th days after infection and were euthanized at the 60th day after infection for isolation of the prefrontal cortex. *A*, representative images of immunofluorescence-based codetection of phosphorylated Tau (p-Tau, *red*) at Ser202 and NeuN (*green*). DAPI was used for nuclear staining. Scale bars correspond to 100 μm. Quantification of fluorescence levels of (*B*) p-Tau and (*C*) the number of NeuN+ cells (mean ± SD, n = 6). *D*, phosphorylated Tau at Ser396 and total Tau were assessed by Western blotting. *E*, quantification of Aβ in brain tissue samples was performed by ELISA directed to mouse Aβ42. Values represent mean ± SD (n = 6). Group means were compared by two-way ANOVA followed by Tukey’s post hoc test; *p* < 0.05 values are embedded in the graphs.

Next, we evaluated the effect of PZQ and NAC/Def over Tau phosphorylation. The increase in Tau phosphorylation observed in the prefrontal cortex of infected animals was not affected by NAC/Def treatment, but PZQ alone or in combination with NAC/Def inhibited Tau phosphorylation to control levels (Fig. 7, *A* and *B*). This effect was confirmed with Tau phosphorylation at Ser396 by Western blot (Fig. 7*D*). Coimmunostaining for the neuronal nuclear protein NeuN demonstrated a significant decrease in the total number of neurons in infected animals, and combined anthelmintic and antioxidant treatment alleviated this effect (Fig. 7, *A* and *C*). These observations led us to investigate another usual marker of neurodegeneration, so we decided to assess amyloid beta peptide (Aβ) accumulation in the prefrontal cortex using a commercial immunoassay kit for quantification of mouse Aβ42 (Fig. 7*E*). ELISA quantification demonstrated that Aβ levels were increased by approximately 7-fold in infected animals. Administration of either PZQ or NAC/Def significantly decreased Aβ accumulation, whereas their combination inhibited Aβ accumulation to control levels (Fig. 7*E*).

**Figure 8:**
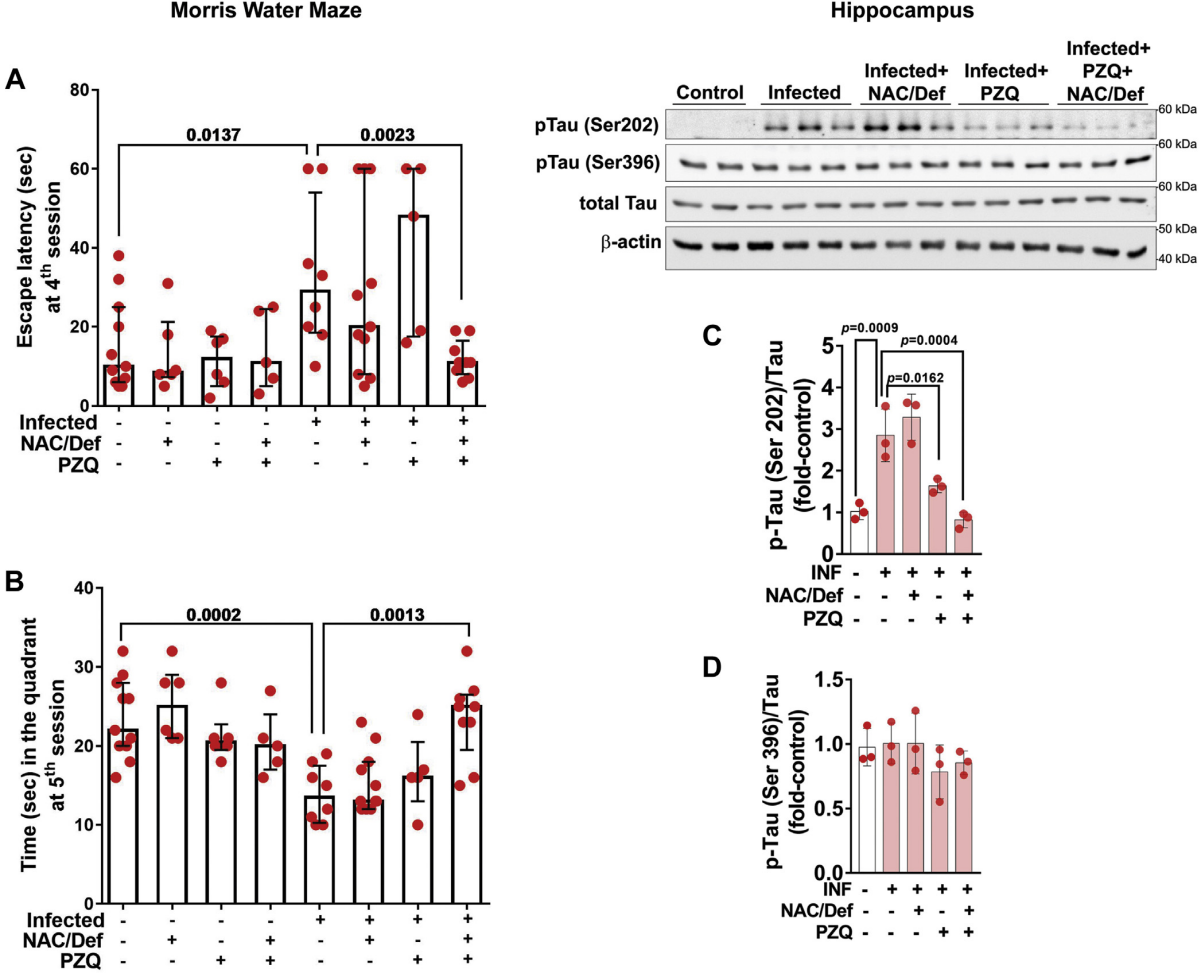
**Effect of anthelmintic and antioxidant treatments over Morris water maze performance and Tau phosphorylation in the hippocampus of mice infected with *S. mansoni*.** Infected mice received daily PZQ (100 mg/kg) and/or combination of NAC/Def (200 mg/kg) from the 42nd to the 46th days after infection. At the 55th day after infection, animals started daily training sessions at the Morris water maze task for four consecutive days. The latency time to escape to the platform was recorded at the fourth session (*A*, 58th day after infection) and, at the fifth session (*B*, 59th day after infection), the time that each animal spent in the quadrant corresponding to the subtracted platform was recorded. Median with interquartile range values are shown. Individual *p* values obtained with one-tailed Mann–Whitney test are shown only when *p* < 0.05. In the hippocampus, WB analyses of the protein Tau phosphorylated at (*C*) Ser202 and (*D*) Ser396 were performed. Mean values ±SD are shown in graphs (n = 3 for WB). Group means were compared by two-way ANOVA followed by Tukey’s post hoc test; *p* < 0.05 values are embedded in the graphs.

Finally, animals were subjected to a Morris water navigation task to evaluate the effect of anthelmintic and antioxidant treatments over spatial learning and memory impairment. After daily training sessions for four consecutive days, the higher latency time to find the platform of the infected group was significantly diminished when infected animals received combined treatment of PZQ and NAC/Def (Fig. 8*A*). On the fifth day, after removal of the platforms, infected animals that received PZQ + NAC/Def displayed an increase in the time spent in the quadrant compared with infected animals that received no treatments (Fig. 8*B*). Differences in swimming speeds were not observed, and animals did not show impairments on motor performance as evaluated by rota-rod test prior to training (data not shown). These results, altogether, indicate that combined anthelmintic and antioxidant treatment can prevent the impairment in spatial learning and memory caused by *S. mansoni* infection, which is not observed with isolated administration of either PZQ or NAC/Def. Given that the hippocampus is directly associated with spatial memory, we also checked the levels of neurodegenerative markers in this structure and observed that the levels of Tau phosphorylated at Ser202 were increased in infected animals, and treatment with PZQ and NAC/Def+PZQ inhibited these effects (Fig. 8*C*), but significant changes in the phosphorylation of Tau at Ser396 were not detected (Fig. 8*D*).

## Discussion

Neuroschistosomiasis is defined as the invasion of *S. mansoni* to the CNS and the neuronal tissue lesions that occur due to the host immune response and granuloma formation triggered by the presence of parasites or eggs (4, 5). Here, we followed animals from 30 to 105 days after infection and then focused further analysis at 60 days after infection, a period that parasite invasion to the CNS is not observed, which was confirmed by histological analysis (Fig. S2*G*). In fact, the Swiss Webster strain is considered an inadequate model to study neuroschistosomiasis, as this condition develops only at late stages of infection and in a very low proportion of animals (20, 21, 22). For such reason, we considered it an ideal model to evaluate the neurological changes that take place in response to systemic infection with *S. mansoni*, which accounts for most schistosomiasis cases. However, some observations on this model must be pointed out: in order to mimic the natural route of infection, mice are used at a young age (5 days old), when they do not have hair to prevent infection through skin. In this context, it may not be ruled out that this model may present unidentified effects over development. Nonetheless, it must also be considered that school-aged children are reportedly more susceptible to *S. mansoni* infection than adults (9, 10), and thus the present model may serve as a parallel for such context.

We observed an increased SOD1 content in infected animals with 30 days of infection, but in later periods SOD1 was increased in both the control and infected groups. This increase in SOD1 levels in control animals is not surprising, as it has been widely discussed that superoxide-dependent signaling in the brain is active but it also may lead to accumulating oxidative damage with aging; thus, oxidative damage markers as well as antioxidant enzymes content are expected to increase in the brain during aging (23, 24). Indeed, several mutations leading to SOD1 oligomerization and loss of function are associated with amyotrophic lateral sclerosis, which reinforces the importance of SOD1 upregulation during aging (25). It is evident that oxidative stress and inflammation play an important role in the neurological changes observed here, which are suggestive of an early neurodegenerative process. The time-course evaluation indicated that the response to oxidative and inflammatory signaling in the brain is highly dynamic and variable with the progression of the infection. We were aware that the period of 39 to 45 days after infection is crucial for the pathogenesis of *S. mansoni*, as the majority of larvae that infected the host had reached maturity and there are several physiological and biochemical manifestations taking place in different organs; notably, we had previously observed oxidative stress, Tau phosphorylation and RAGE upregulation (17). Taking this into account, we decided to start the 5-day administration of PZQ and NAC/Def at the 42nd day after infection, in order to eliminate adult worms during the beginning of egg laying into the host and then to analyze animals at the 60th day post infection. This experimental design allowed us to study the impairment in spatial learning and the evolution of neuroinflammation, oxidative stress, and neurodegenerative markers at an early stage of the disease and evaluate the recovery capacity of the brain after anthelmintic treatment. The rationale for examining animals 2 weeks post drug treatment was based on the time points of the effects observed in brain, the mechanism of effect of the anthelmintic drug, and the life cycle of the parasite. We have chosen 60 days after infection to analyze biochemical and morphological changes (as well as behavioral effects) as this time point was the earliest with Tau phosphorylation at both Ser202 and Ser396 in the time course experiment. The administration of PZQ was performed between the 42nd and the 46th days after infection because this is the period when worms start to lay eggs in the host, so we wanted to avoid “background noise” effects from egg-induced inflammation or newborn worms from hatched eggs in treated groups (NAC/Def treatment was administered along with PZQ).

The increase in circulating inflammatory mediators is well documented for schistosomiasis, and we confirmed that several proinflammatory cytokines were increased in the serum at the 60th day post infection. Nonetheless, the systemic response to infection is highly variable according to the stage of the disease and life cycle of the parasite, and this response may present a variable profile on different days after infection. Consistent with most observations in this study, the combination of PZQ and NAC/Def was more effective in inhibiting the increase in proinflammatory mediators compared with isolated administration of either PZQ or NAC/Def. The observation that only the combination of all drugs used in this study was able to significantly inhibit IFN-γ, TNF-α, and MCP-1, which along with IL-12 were increased in serum of infected animals, is important to understand the causes of the changes occurring in brain and behavior. As parasites are not present in the CNS at this stage, the changes observed in the prefrontal cortex probably result as a consequence of the systemic response to infection and possibly are dependent on cellular responses evoked by IFN-γ, TNF-α, and MCP-1. Of interest, the levels of TNF-α in the brain were not affected by infection, whereas the circulating levels of this cytokine were increased 60 days after infection, as observed in Figure 4*A*. It is well known that circulating TNF-α levels are strongly influenced by liver inflammation in schistosomiasis, but our results indicate that the tissue levels of this cytokine are not influenced at this stage of infection or by its increase in serum. However, we did not check the levels of cytokines in the cerebrospinal fluid (CSF). In an acute LPS model of systemic inflammation, TNF-α and IL-1β are increased in both serum and CSF, but not in brain tissue (26). Thus, it is possible that TNFα and other cytokines may trigger proinflammatory signals in the brain even if they are not expressed locally, but to answer this question the levels of proinflammatory modulators in CSF should be examined in detail. In schistosomiasis, the host immune system deals with several different phases of the parasite life cycle, each one expressing a myriad of antigens that provoke different humoral and cellular responses (27). Characteristically, such responses present great variation according the parasite cycle; during transition from cercariae to schistosomula, and then to adult worms and the eggs produced by them, some immune responses increase continuously, whereas others are inhibited (13). However, in any scenario, phenotypic changes in astrocytes and microglial cells only occur in response to disturbances in the homeostasis of the CNS.

Both astrocytes and microglia underwent relevant changes in infected animals and in treated groups. Not only were the immunostaining levels of GFAP and Iba-1 increased but also each type of treatment resulted in distinct phenotypic alterations, with particular morphological changes (Figs. S3 and S4, Table 1 and Fig. 5). Of importance, phenotypic changes were observed even when the levels of GFAP and Iba-1 did not demonstrate significant differences among groups. In animals treated only with PZQ, microglial cells presenting a morphological pattern known as “jellyfish,” which is associated with activated cells with high phagocytic activity (28), were observed. This morphological pattern is often observed in brain trauma and other types of injuries not associated with pathogen infection and that results in the increase in local cell debris. Consistent with this, astrocyte “scars” were observed in infected animals treated with PZQ. Furthermore, the analysis of specific parameters related to cell activation revealed that PZQ and NAC/Def caused different responses in astrocytes and microglia. Infection with *S. mansoni* increased the cell number of both cell types, but only the combination of PZQ and NAC/Def inhibited this effect in astrocytes, whereas all treatments were able to revert this effect in microglia cells. Besides, the infection also enhanced the soma eccentricity and decreased the cell extent in astrocytes. Astrocytic processes form the tripartite synapse with neurons; thus, the decrease in such processes could, in turn, decrease glutamate uptake from the synaptic cleft thus enhancing excitotoxicity. This decrease in cell extension was reversed by PZQ alone, suggesting that this effect is a more direct consequence of proinflammatory changes than oxidative stress. Of interest, cumulative threshold spectra analysis demonstrated that, at thresholds between 240 and 250 PIs, PZQ alone further increased the percentage of material thresholded observed in infected animals, causing a left-shift of the Infected+PZQ curve that was even more intense than in the Infected curve (Fig. 5*A*). This effect must be related to increased cell number, since PZQ alone did not reverse the effect of *S. mansoni* infection (Table 1), and also it is reasonable to suggest that the effect of PZQ on the shape and darkness of astrocyte’s processes may be associated with this result, as cell extent was also affected by PZQ in infected animals. In microglia, the increased cell number was reversed by all treatment options (Table 1), but in Iba-1 fluorescence quantification only NAC/Def alone had a statistically significant inhibitory effect (Fig. 4*B*). On the other hand, in cumulative threshold analysis either PZQ or NAC/Def alone inhibited the effect of *S. mansoni* infection, but their combination had no effect at all (Fig. 5*B*). Of interest, the combination of NAC/Def increased cell solidity and cell extent compared with infected animals, although a significant effect of infected animals compared with control in such parameters was not detected (Table 1). The cumulative threshold spectra analysis is a particularly useful approach as it distinguishes the differences between groups at different thresholds, thus avoiding the possibility of employing a threshold that could “mask” the differences. However, it is difficult to establish the effect of NAC/Def and PZQ over microglial response considering the present data altogether. It seems that the antioxidant treatment has a more prominent effect over Iba-1 immunostaining levels, but the inhibitory effect of PZQ was more consistent in the cumulative threshold spectra analysis as it was the only one presenting effects at two different PIs (Fig. 5*B*). This is particularly intriguing, as we would expect that antioxidant treatment had a more consistent effect over parameters of microglial activation due to the role of NADPH oxidase-driven reactive species in this process.

Assessment of oxidative/nitrosative damage markers and Nrf2 indicates that PZQ does not enhance oxidative stress in the prefrontal cortex of infected mice. In fact, combination of NAC/Def with PZQ treatment seemed to prevent both oxidative damage and glial activation. Moreover, the content of the neuronal protein NeuN, ubiquitously expressed in neurons, is decreased in infected animals, and PZQ treatment did not cause further loss of NeuN-positive cells. Altogether, these data may indicate a new therapeutic possibility to be explored in order to preserve neural integrity in schistosomiasis.

Here, we tested for Tau phosphorylation at Ser202 and Ser396 sites, and both sites had increased phosphorylation in infected animals. Phosphorylation of Tau at Ser202 has been well characterized in AD, where staging of the disease is based on labeling of Ser202/Thr205 phosphorylation; increased phosphorylation at these sites is associated with an early degenerative modification of the cytoskeleton (29, 30). Phosphorylation of Ser396 was reported as one of the earliest events associated with neurofibrillary tangles formation in AD and Down’s syndrome (31), although it was commonly associated with late stages of AD owing to its more pronounced presence in neurofibrillary tangles (32). Infected animals presented a steady increase in Tau phosphorylation at both sites over time, and our data clearly indicate that this effect is not dependent on oxidative stress, as isolated NAC/Def treatment did not affect phosphorylation at both sites. Isolated anthelmintic treatment, however, had a strong inhibitory effect over Tau phosphorylation, indicating that the presence of the parasite in the body is essential for this effect. Combination of PZQ and NAC/Def inhibited Tau phosphorylation to control levels, suggesting that antioxidant treatment ameliorates the protective effect of PZQ. Since parasites are not present in the brain in these experiments, and considering the lack of effect of NAC/Def, it is highly likely that Tau phosphorylation is a result of peripheral proinflammatory signaling triggered by the parasite. Alterations in Tau phosphorylation associated with neurotoxic and/or neurodegenerative processes have been previously observed in different states of systemic inflammation, such as LPS-induced endotoxemia (26) and polymicrobial sepsis (33).

Systemic inflammation has also been associated with Aβ accumulation in the CNS (33, 34). To our knowledge, this is the first observation of brain Aβ accumulation in schistosomiasis. In AD, neurodegeneration is associated with the formation of senile plaques, the histopathological structures formed by aberrant Aβ deposition and characteristic of the late stages of the disease. Although we observed a marked increase in Aβ tissue levels here, the limitations of the rodent model do not allow one to hypothesize any parallel with human neurodegenerative processes. As an intrinsic characteristic of the animal model, Swiss Webster wild-type mice do not develop senile plaques. Notwithstanding, in genetically modified models where such changes are observable, they are detectable only with advanced neurodegeneration, which is not reasonable to be expected at this stage of schistosomiasis (60 days after infection). Besides that, although neurological impairment is described for schistosomiasis, no clinical observations of a rapid evolving neurodegenerative process have been reported so far. In AD, senile plaques are thought to develop in the course of years or decades, probably resulting from a combination of causative factors, which are highly likely to involve changes in Aβ precursor protein processing in vulnerable neurons, enhanced γ-secretase activity, and Aβ formation and accumulation (35). Nonetheless, the marked accumulation of Aβ, along with the increase in Tau phosphorylation, neuroinflammation, and oxidative stress observed here, presents a context that is consistent with the hypothesis that systemic inflammation induced by *S. mansoni* infection affects the CNS integrity and triggers biochemical changes in the prefrontal cortex that might result in a long-term process of neurological impairment. Our behavioral data also corroborate this hypothesis.

The impaired performance of infected animals at the Morris water maze task correlates well with the clinical observations of impaired cognitive function and attention levels in schistosomiasis. Overall, the results of this test suggest that infected animals have a decreased capacity of learning and spatial memory acquisition, since this group presented poor scores of learning during the first three training session days (data not shown) and also in the fourth training session day. In another batch of experiments not included in the present work, we did not detect changes in the rota-rod test, excluding the possibility of motor changes, and also did not observe modifications in grooming behavior, which could suggest modifications in the state of anxiety or stress (data not shown). The results at the Morris water maze were also consistent with our data in all other assays, in the sense that only the combination of anthelmintic and antioxidant treatments was effective in inhibiting the impairment caused by *S. mansoni* infection. Thus, although Tau phosphorylation and Aβ accumulation are not defining indicators of a neurodegenerative process, a context also involving increased neuroinflammation and oxidative stress, as well as impaired spatial learning and memory, compelled us to suggest that a relevant (and, probably, long-termed) detrimental process is induced in the brain of mice infected with *S. mansoni*, even without the presence of parasites in the CNS. Moreover, as the results of the Morris water maze also suggested that the hippocampus was affected by *S. mansoni* infection to some degree, we evaluated some of the parameters of neurodegeneration in this region, and the results indicate that, although Tau phosphorylation at Ser202 is increased, a more detailed analysis of this structure should be conducted to better understand the extent at which neurodegenerative modifications are taking place. It also remains to be clarified if the injuries in prefrontal cortex and hippocampus may evolve to serious neurological complications at long term, but our results indicate that they are reversible if an adequate anthelmintic and antioxidant treatment is applied at early stages of infection.

**Figure 9:**
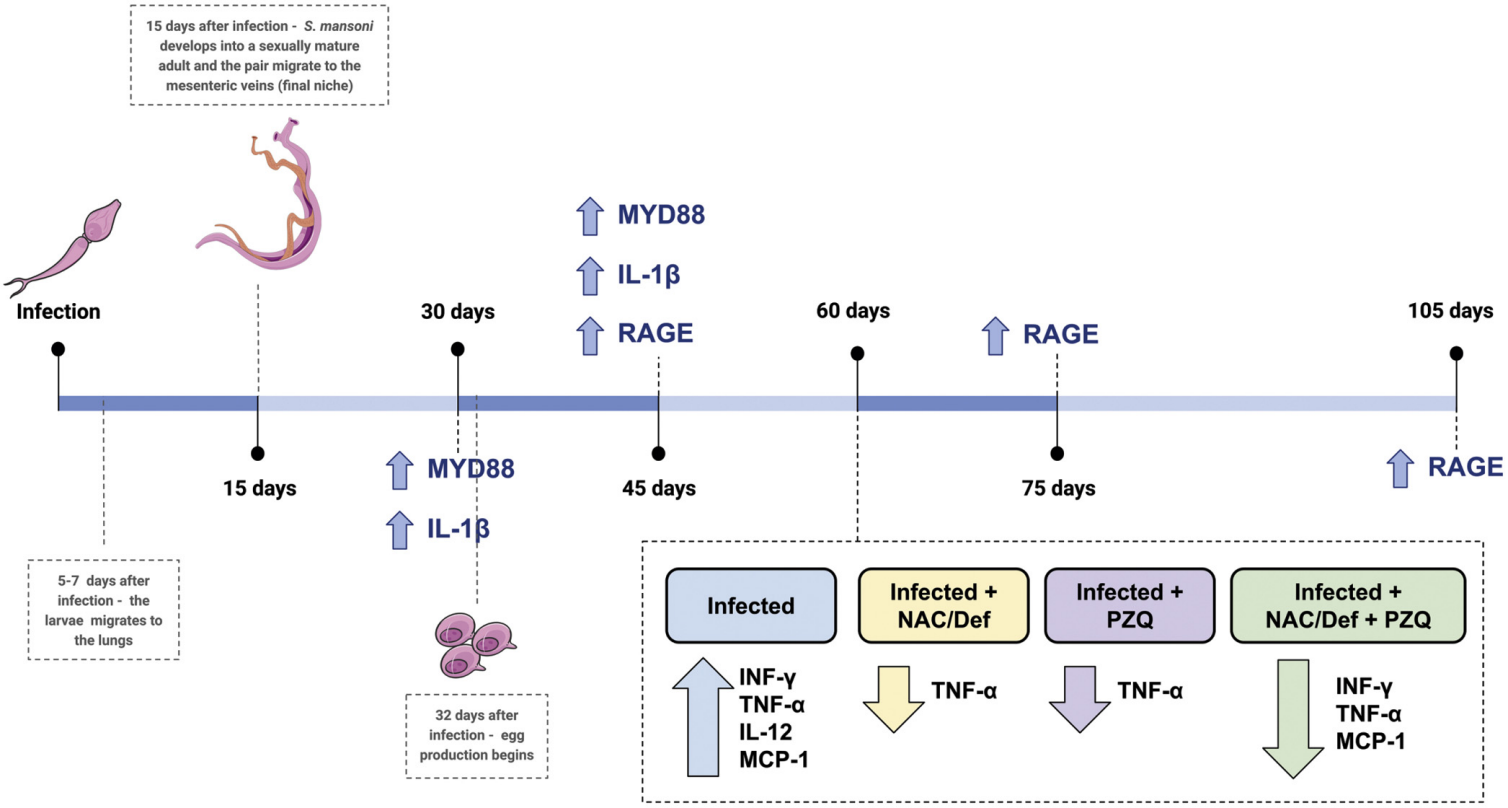
**Changes in cytokines and chemokines along the progression of *S. mansoni* life cycle in the host.** The changes at 60 days after infection were observed in serum, whereas changes indicated in all other time points were observed only in the brain.

The detailed mechanism of the molecular events connecting systemic *S. mansoni* infection with the brain and behavioral changes observed here remains to be experimentally elucidated. Our data indicate that both inflammation and oxidative stress play important roles in the neurological changes observed here, but it is difficult to postulate if either proinflammatory signaling or redox-mediated signaling is the main drive of CNS homeostasis disruption. One suitable approach to address this could be network-based analysis of transcriptional data obtained from the prefrontal cortex and from peripheral tissues/cells that contribute to the systemic response against *S. mansoni* infection. To develop such discussion, we conducted a search for transcriptional data in public databases and did not find available datasets of the CNS from human patients or animal models infected with *S. mansoni*. Nonetheless, transcript datasets from liver (RNA-seq, GSE94132) from mice at postinfection day 32 and T-effector (Teff) and T-regulatory (Treg) splenic immune cells (microarray, GSE17580) from mice at postinfection day 63 were available, so we collected these data and conducted a network-based study of differential expression followed by gene ontology analysis. Our analysis of liver RNA sequencing (RNA-seq) data identified 205 upregulated and 74 downregulated genes, which were used to build a protein–protein interaction network (Fig. S6*A*). Briefly, differentially expressed genes (DEGs) were organized in cohesive clusters of up- and downregulated genes. Ontology analysis revealed that catabolic processes such as digestion, lipid catabolism, and proteolysis were diminished in the liver of infected mice, whereas immune processes, defense mechanisms, and stress response were increased, with immune system responses presenting the highest increase (Fig. S6, *B* and *C*). Analysis of the GSE17580 dataset revealed 164 DEGs for Teff cells (102 up and 62 down) (Fig. S7, *A*–*C*) and 44 up- and 47 downregulated genes for Treg cells (Fig. S8, *A*–*C*). The Treg ontology showed suppression of Ras, ARF, and GTPase modulation (Fig. S8*B*). However, immune-related pathways such as regulation of MHC class II, immune response, and cytokines production were increased in Treg cells (Fig. S8*C*). Effector T cells (Teffs) also showed an increase in immune system pathways: immune response, regulation of cytokines production, and modulation of acute inflammatory response (Fig. S7*C*). IFN-γ was upregulated both in Treg and Teff cells, corroborating with our serum results. An increase in the expression of cytokines (TNF-α and IL-12) that interact with INF-y was evidenced in serum, but enhanced IL-10 expression was not detected in our serum analysis (Fig. 4*A*). This is a preliminary analysis performed with a limited collection of data, but they indicate a preponderance of immune system–related processes in the systemic response against *S. mansoni*, which is suggestive that oxidative stress is mostly a by-product of immune activation in schistosomiasis. Although our experimental data indicate that antioxidant treatment may be a beneficial approach to counteract neurological damage caused by systemic infection with *S. mansoni*, this analysis reinforces the role of the systemic immunologic response in the etiology of neuroinflammation, neural damage, and cognitive impairment in schistosomiasis. In Figure 9, a summary of the changes in cytokines and chemokines observed along the progression of *S. mansoni* life cycle in our experimental model is presented.

Based on the present data, we conclude that systemic inflammation caused by *S. mansoni* infection leads to neuroinflammation and oxidative stress in the prefrontal cortex, which are associated with biochemical changes characteristic of neurodegenerative diseases: Tau phosphorylation at Ser202 and Ser396 and Aβ accumulation. In the hippocampus, Tau phosphorylation at Ser202 is observed as well. These changes are also associated with impaired spatial learning and memory. It remains to be answered whether chronic schistosomiasis can trigger a typical long-term neurodegenerative process and if our observations are paralleled in human cases. Our results also indicate that antioxidant therapy, combined with the recommended PZQ administration, may be useful to reverse these detrimental effects to the brain.

## Experimental procedures

### Chemicals

Electrophoresis and immunoblotting apparatus were from Bio-Rad and GE Healthcare Brazilian Headquarter, respectively. Detailed antibodies information (source, catalog number, and working dilution) is provided in Supporting information (Table S1). All other reagents used in this study were of analytical or HPLC grade.

### Animals and infection

Swiss Webster male mice, provided by the Instituto de Ciência e Tecnologia em Biomodelos (ICTB) of FIOCRUZ were housed under 12-h light/dark cycle. Animals had free access (*ad libitum*) to water and commercial food (Chow Nuvilab CR-1 type). Five-day-old male mice (still lacking hair) were infected by exposure of the skin and tail, mimicking the natural route of infection, by 150 ± 10 cercariae of *S. mansoni* (Belo Horizonte strain) during 30 min under incandescent light. The infection was confirmed by counting worms in the mesenteric cavity (±35). In time course experiments control (uninfected) and infected animals were euthanized 30, 45, 60, 75, and 105 days after infection. Animals were divided into groups of 6 to 8 mice and randomly used for the different analyses (n = 3 for Western blot, n = 4 for ELISA, and n = 4–8 for parasitological and physiological analyses). To evaluate the effect of anthelmintic and antioxidant treatments, infected animals were treated with the anthelmintic praziquantel (PZQ) and the antioxidants *N*-acetyl-L-cysteine (NAC) and deferoxamine (Def). The compounds were administrated daily, by gavage, from the 42nd to the 46th day after infection, and euthanasia was performed on the 60th day post infection. NAC and Def were administered at 200 mg/kg each, whereas PZQ was administered at 100 mg/kg (see Figs. S1 and S2 in Supporting information for graphical design of experiments). Only male mice were used in this study. For time course experiments, each group had six animals, and for drug treatment experiments, each group started with 12 animals (control and infected groups started with 14 animals each). After Morris water maze tests, animals were divided for the measurement of the different biochemical, physiological, and immune parameters as follows: n = 6 for Western blot, n = 6 for immunofluorescence analysis, n = 6 for Multiplex-based cytokine analysis.

### Morris water maze

Behavioral procedures were conducted between 8 and 11 AM in a sound-isolated room. Before every experiment session, the animals were acclimated for 30 min in the room. At the 55th day after infection, training sessions were started. For the training sessions, once a day, for 60 s, mice were placed individually in a pool (1 m diameter) containing a submerged (not visible) platform in one of its quadrants. In the first training session, when the mouse found the platform before 60 s, it was left to remain on the platform for 5 s and then was gently removed from the water and returned to the home cage. When the mouse did not find the platform, it was placed on the platform for 20 s and then returned to the home cage. The subsequent training sessions were performed once a day, for two consecutive days. At the second and third training sessions, when the mouse found the platform before the 60-s cutoff, the time was recorded and the animal was returned to the home cage. When the mouse did not find the platform, the time was recorded as 60 s, and the animal was returned to the home cage. At the fourth day, (recorded training session), the time to reach the platform was registered for each mouse. The mouse that found the platform before the 60-s cutoff was placed in the home cage and the time was recorded. The mice that did not find the platform were removed from the water and the time was recorded as 60 s. At the fifth day (test session), the platform was removed and the time that the animal spent swimming in the platform quadrant was recorded (36). Animals did not show differences on motor performance as evaluated by rota-rod test prior to training (37).

### Tissue samples

Mice were inhaled with CO_2_ and immediately submitted to the perfusion procedures while cardiac activity was still evident. Toe-pinch reflex was checked and, if not responsive, an incision through the abdomen was performed at diaphragm length, and then a cut of the rib cage up to the collarbone on both sides of the ribs was made for best view of the heart. An incision in the posterior end of the left ventricle was performed to insert an olive-tipped perfusion needle through the ventricle to extend straight up about 5 mm, and another incision in the right atrium was performed to allow an opening for flow of the solution. The needle was stabilized, and the descendent aorta was clamped to optimize perfusion in the CNS with a hemostat. The blood was collected and centrifuged at 2000*g* for 15 min to separate the serum. After blood sampling, each group was divided into two, n = 6 for Western blotting and n = 6 for immunofluorescence microscopy and tissue isolation followed as described below.

### Western blot

Brains were carefully removed, and the prefrontal cortex was isolated using the Rodent Brain Matrix (RBM-4000C, ASI instruments). A mouse brain atlas (38) was used to identify the following coordinates: Bregma 3.08 mm to Bregma 2.58 mm. The tissue samples were stored at −20 °C. To prepare samples for Western blotting a RIPA buffer-based protocol (39) was used to homogenize the tissue. Laemmli sample buffer was added to complete the volume after protein content determination by Bradford method. Equal amounts of protein samples (30 μg/lane) were fractioned by SDS-PAGE and electroblotted onto nitrocellulose membranes in a Trans-Blot SD Semi-Dry Electrophoretic Transfer Cell (Bio-Rad). Electroblotting efficiency was checked with Ponceau S staining. Tris-Tween saline solution (TTBS, 100 mM Tris-HCl, pH 7.5, containing 0.9% NaCl, and 0.1% Tween-20) was used to wash the membranes and to dilute primary and secondary antibodies. The primary antibodies incubations were performed according to the manufacturer's instructions of each antibody. Anti-rabbit or anti-mouse IgG peroxidase–linked secondary antibody was incubated with membranes for 2 h, and the immunoreactivity was detected by enhanced chemiluminescence using Supersignal West Pico Chemiluminescent kit from Thermo Fisher Scientific. Molecular weight was accessed with a biotinylated protein ladder (5 μl per lane). The chemiluminescence was captured with an ImageQuant LAS 4000 (GE Healthcare). Densitometry analysis of the images was performed with ImageJ software (ImageJ version 1.49, National Institutes of Health). Blots were developed to be linear in the range used for densitometry. All results were expressed as a relative ratio to β-actin or to their respective total protein content. Six animals per group were used for statistical analysis. The antibodies description is detailed at Table S1.

### Perfusion fixation and immunofluorescence histology

Following blood sampling, six animals per group were perfused (flow rate 20 ml/min) with 0.9% sterile saline over 10 min followed by 10 min with paraformaldehyde solution 4% in PBS (pH 7.4). The brains were removed and maintained in 4% paraformaldehyde for 24 h at 4 °C, then transferred to sucrose 15% for 24 h at 4 °C, and finally transferred to sucrose 30% for 24 h at 4 °C. After rapid drying, samples were frozen at −20 °C. Twenty-four hours later the prefrontal cortex was sectioned into slices of 30 μm on the coronal plane using a cryostat at −20 °C (Jung Histoslide 2000R; Leica). A total of 5 to 7 slices per mouse were collected in PBS containing 0.1% Triton X-100 (PBS-0.1%). The free-floating sections were saturated with 5% albumin for 2 h to block nonspecific binding. The primary antibody was incubated for 48 h at 4 °C. Anti-Iba-1, anti-GFAP, anti-Nrf2, anti-phospho-Tau, anti-4-HNE, and anti-nitrotyrosine were used in PBS with 2% bovine serum albumin. Anti-4-HNE and anti-nitrotyrosine were diluted in PBS with 2% bovine serum albumin. DAPI was used for nucleic acid staining. After washing 4× with PBS-0.1%, tissue sections were incubated with secondary antibody according to IgG species in PBS with 2% bovine serum albumin. After 1 h at room temperature, the slices were washed several times in PBS-0.1%, transferred to gelatinized slides, mounted with FluorSaveTM (345789, Merck Millipore), and covered with coverslips. The images were obtained with a Microscopy EVOS FL Auto Imaging System (AMAFD1000, Thermo Fisher Scientific). Quantitative analysis of immunofluorescence staining was performed with ImageJ software (ImageJ version 1.49, National Institutes of Health). The mean was calculated using five slices from each region per animal. Six animals per group were used for statistical analysis.

### Digital reconstruction of astrocytes and microglia for morphological analysis

Morphological reconstruction for the analysis of cellular parameters of astrocytes and microglia was performed with Matlab v2013b as described by Kongsui *et al.* (40). The marked cells were segmented from the background with the multilevel Otsu threshold, a method that calculates the limits to minimize the variation in pixel intensity interclass between the various classes (soma, processes, and background). Different characteristics of the cells, such as cell number, primary branch, total number branch, total branch length, cell radius, soma area, soma eccentricity, cell area, cell solidity, and cell extent were analyzed with Matlab. These morphological parameters were automatically calculated using the Matlab image processing toolbox function, according to the description in (40).

### Cumulative threshold analysis

Cumulative threshold analysis was performed using Matlab custom script as described in (40, 41). For each of the acquired images, the number of pixels occurring at each of the pixel intensities was determined. The pixel intensity (PI) values are then rank ordered 0 to 255 along with the corresponding number of pixels that occur at each value. Then a determination of the cumulative percentage of pixels is performed for the range of PI values. For statistical analysis, the cumulative threshold spectra scores for each picture from each of the groups were calculated. Group differences were assessed at different number of PI values that represented different components of the signal.

### Serum cytokines determination

Serum of mice was assayed for the presence of the following inflammatory mediators: interferon-gamma (IFN-γ), interleukin (IL)-10, tumor necrosis factor-alpha (TNF-α), IL-1β, IL-6, IL-12, Monocyte Chemoattractant Protein-1 (MCP-1), and keratinocyte-derived chemokine (KC) was obtained and the assay performed in accordance with the manufacturer's instructions and were collected using Multiplex Bio-Plex system (Bio-Rad). Data analyses for all assays were performed using the Bio-Plex Manager software (42).

### Indirect ELISA

The oxidative marker 3-nitrotyrosine (or, simply, nitrotyrosine) was measured in mice prefrontal cortex. Samples were homogenized in PBS and incubated in ELISA plates for 24 h at 4 °C under gentle shaking. The plates were then washed three times with TTBS. Primary and secondary antibodies were incubated according manufacturer's instructions. A substrate solution (TMB ELISA spectrophotometric detection kit from BD Biosciences) was added to each well for secondary antibodies detection, and the reaction was carried out within 15 min at 37 °C. Sulfuric acid (12 M) was used as a stop solution. ELISA plates were read at 450 nm in a spectrophotometer.

### Aβ quantification

Detection of Aβ in brain tissue samples was performed using a commercial ELISA kit for quantification of mouse Aβ42 (Invitrogen, Cat. #KMB3441) following the instructions of the manufacturer. Briefly, brain samples were homogenized with cold 5 M guanidine-HCl/50 mM Tris, pH 8.0. The samples were diluted with cold PBS with 1× protease inhibitor cocktail (Sigma Cat. # P-2714) and centrifuged at 16,000*g* for 20 min at 4 °C. The supernatants were transferred into clean microcentrifuge tubes and kept on ice. The protein normalization was performed with Bradford assay (2.5 μg/μl). The samples and standards were platted, and the ELISA procedure was performed rigorously according the kit protocol. The results are expressed as pg/mg of protein.

### Protein assay

Total protein was quantified by Bradford assay and used to normalize all data (43).

### Transcripts data collection, networks generation, and gene ontology analyses

To understand the influence of systemic *S. mansoni* infection over the CNS, we conducted an analysis of transcription data aiming to identify the changes in gene expression of tissues affected by the parasite that are most likely to be responsible for the changes observed at the CNS level. Transcription data were collected from: 1) mouse liver at 32 days post infection, RNA-seq data accession is GSE94132 (28650976); 2) T effector (Teff) and T regulatory (Treg) cells extracted from mouse spleen at 63 days post infection, microarray data accession is GSE17580 (20007528). DEGs were considered to have an FDR <0.05 and the base 2 logarithmic fold change correction (logFC) major or equal to ±1. Gene expression analyses of RNA-seq data were performed with R with the package’s edger (19910308) and limma (25605792), and microarray data were analyzed with GEO2R tool. Protein–protein interaction networks were generated using the DEGs as seeds in STRING v.10.5 (25352553). The following parameters were utilized in STRING: all active interaction sources, excluding textmining; 0.400 as minimum required interaction score; no more than 20 neighbors in the first shell; and none for second shell. In addition, network layout and analyses were made in Cytoscape v. 3.7.0 (14597658). The counting of the number of neighbors to each protein was made using Cytoscape's plugin CentiScaPe v.2.2 with the degree parameter (26594322). All GO analyses were made on Cytoscape's plugin BiNGO 3.0.3 (15972284). The parameters set on BiNGO were hypergeometric test for statistic distribution, FDR correction, 0.05 for significance level, biological process, and *Mus musculus* as organism.

### Statistics

Statistical analysis was performed using GraphPad Prism version 7 (GraphPad Software Inc). The results were expressed as mean ± standard deviation (SD). Differences were considered significant when *p* < 0.05. Group means were evaluated by multiple *t* tests with correction for multiple comparisons with Holm–Sidak (time-course experiments) and two-way ANOVA followed by Tukey’s post hoc test (experiments with multiple groups). For behavioral analyses, individual groups were compared by the Wilcoxon tests, and comparisons among groups were performed using the Mann–Whitney U test.

### Study approval

All procedures were according to the animal care standards of the Committee of Ethics for the Use of Animals (CEUA) of the Universidade Federal do Rio Grande do Sul (UFRGS); The Guide for the Care and Use of Laboratory Animals from National Institutes of Health (2011), and the guidelines established by the Ethics Committee for Animal Use of Fundação Oswaldo Cruz (FIOCRUZ). The study was registered at FIOCRUZ (CEUA, license number L044/2015).

## Data availability

All data are included in the article.

## Supporting information

This article contains supporting information.

## Conflicts of interest

The authors declare that they have no conflicts of interest with the contents of this article.
