# Supplementary material for: Neurological impairment caused by Schistosoma mansoni systemic infection exhibits early features of idiopathic neurodegenerative disease
Source: J Biol Chem. 2021 Jul 22;297(2):100979. doi: 10.1016/j.jbc.2021.100979 (PMC8361297; doi:10.1016/j.jbc.2021.100979)
Supplement: Supplemental Figures S1–S8 and Table S1 [file mmc1.pdf]

SUPPORTING INFORMATION

Neurological impairment caused by *Schistosoma mansoni* systemic infection exhibits early features of idiopathic neurodegenerative disease

Juciano Gasparotto, Mario Roberto Senger, Emilio Telles de Sá Moreira, Pedro Ozorio Brum, Flávio Gabriel Carazza Kessler, Daniel Oppermann Peixoto, Alana de Castro Panzenhagen, Lin Kooi Ong, Marlene Campos Soares, Patricia Alves Reis, Giuliana Viegas Schirato, Walter César Góes Valente, Bogar Omar Araújo Montoya, Floriano Paes Silva-Júnior, José Claudio Fonseca Moreira, Felipe Dal-Pizzol, Hugo Caire de Castro-Faria-Neto, Daniel Pens Gelain

List of materials:

Table S1. Detailed antibody information
Figure S1. Infection with *Schistosoma mansoni* is confirmed by evaluation of worms in the mesentery and analysis of liver and spleen parameters
Figure S2. Effect of anthelmintic and antioxidant treatments over somatic parameters of disease and confirmation of parasite absence in the brain of mice infected with *Schistosoma mansoni*
Figure S3: Protein phosphorylation normalized to β-actin content.
Figure S4: Morphological observation of GFAP immunofluorescence staining in prefrontal cortex of mice infected with *S. mansoni*
Figure S5. Morphological observation of Iba-1 immunofluorescence staining in prefrontal cortex of mice infected with *S. mansoni*
Figure S6. Transcription data collected from mouse liver at 32 days post-infection, RNA-seq data accession is GSE94132 (28650976)
Figure S7. Transcription data collected from T effector (Teff) cells extracted from mouse spleen at 63 days post-infection, microarray data accession GSE17580 (20007528)
Figure S8. Transcription data collected from T regulatory (Treg) cells extracted from mouse spleen at 63 days post-infection, microarray data accession GSE17580 (20007528)

Table S1. Detailed antibody information. WB- Western blotting. IF- immunofluorescence.

Antibody	Work dilution	Catalog number	Source
**4-HNE**	ELISA - 1:5000<br>IF – 1:200	Ab46545	Abcam® (UK)
**Akt**	WB - 1:1000	9272	Cell signalling Technology (USA)
**Alexa Fluor® Plus 488 mouse**	IF – 1:500	A32723	Thermo Fisher Scientific (USA)
**Alexa Fluor® Plus 488 rabbit**	IF – 1:500	A32731	Thermo Fisher Scientific (USA)
**Alexa Fluor® Plus 555 mouse**	IF – 1:500	A32727	Thermo Fisher Scientific (USA)
**Alexa Fluor® Plus 555 rabbit**	IF – 1:500	A32732	Thermo Fisher Scientific (USA)
**Anti-Mouse – peroxidase**	WB – 1:2000<br>ELISA – 1:1000	AP124P	Merck (USA)
**Anti-Rabbit – peroxidase**	WB – 1:2000<br>ELISA – 1:1000	Ap132P	Merck (USA)
**Catalase**	WB – 1:800	14097s	Cell signalling Technology (USA)
**DAPI**	5 µg/mL	D9542	Merck (USA)
**ERK 44/42**	WB – 1:1000	CS9102	Cell signalling Technology (USA)
**GFAP**	IF – 1:500	G4546	Merck (USA)
**GSK-3α**	WB – 1:500	9338	Cell signalling Technology (USA)
**GSK-3β**	WB – 1:500	5558	Cell signalling Technology (USA)
**Iba-1**	IF – 1:500	019-19741	Fujifilm Wako Chemicals (USA)
**IL-1β**	WB- 1:1000	ab9722	Abcam® (UK)
**JNK**	WB- 1:1000	sc-571	Santa Cruz Biotechnology, Inc. (USA)
**MyD88**	WB- 1:1000	4283	Cell signalling Technology (USA)
**NeuN**	IF-1:500	MAB377	Merck (USA)
**Nitrotyrosine (3-NT)**	ELISA – 1:5000<br>IF – 1:200	ab7048	Abcam® (UK)
**Nrf2**	IF- 1:200	12721s	Cell signalling Technology (USA)
**p- GSK-3α/β (Ser21/9)**	WB – 1:500	9331	Cell signalling Technology (USA)
**p-38**	WB – 1:1000	8690	Cell signalling Technology (USA)
**p-Akt (Ser473)**	WB – 1:800	4051	Cell signalling Technology (USA)
**p-ERK 44/42**	WB – 1:1000	4370	Cell signalling Technology (USA)
**p-ERK 44/42**	WB – 1:800	CS9101	Cell signalling Technology (USA)
**p-JNK (Thr183/Tyr185)**	WB – 1:800	9251	Cell signalling Technology (USA)
**p-p38 (Thr180)**	WB – 1:800	9215	Cell signalling Technology (USA)
**p-Tau (Ser202)**	WB – 1:800	39357	Cell signalling Technology (USA)
**p-Tau (Ser396)**	WB – 1:800<br>IF -1:200	9632	Cell signalling Technology (USA)
**RAGE**	WB – 1:2000	ab37647	Abcam® (UK)
**SOD1**	WB – 1:1000	ab13498	Abcam® (UK)
**SOD2**	WB – 1:1000	13194	Cell signalling Technology (USA)
**Tau (Tau46)**	WB – 1:1000	4019s	Cell signalling Technology (USA)
**TNF-α**	WB – 1:2000	ab6671	Abcam® (UK)
**β-actin**	WB – 1:1000	A1978	Merck (USA)

Figure S1

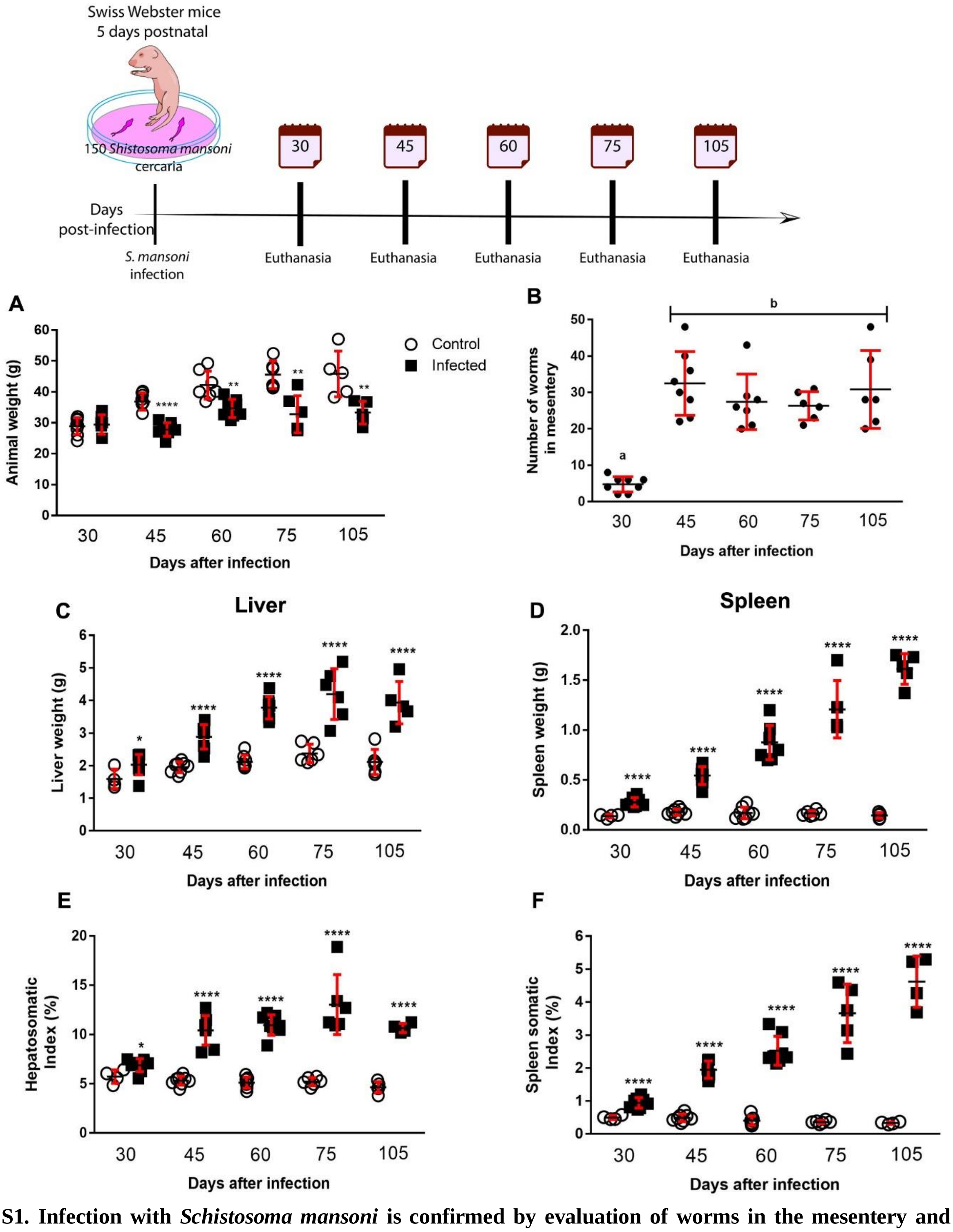

Figure S1. Infection with *Schistosoma mansoni* is confirmed by evaluation of worms in the mesentery and analysis of liver and spleen parameters. Five-days-old mice were infected by exposure of the skin and tail, mimicking the natural route of infection, by 150 ± 10 cercariae of *S. mansoni* (Belo Horizonte strain) during 30 minutes under incandescent light. The infection was confirmed by counting worms in the mesenteric cavity (±135). In time course experiments, control (uninfected) and infected animals were euthanized 30, 45, 60, 75 and 105 days after infection. Somatic and parasitological parameters were evaluated: **A)** animal weight, **B)** liver weight, **C)** hepatosomatic index (liver weight/animal weight*100), **D)** spleen weight, **E)** spleen-somatic index (spleen weight/animal weight*100), **F)** number of worms recovered from mesentery. Control and infected groups were compared with multiple t-test. Asterisks denote significance degrees for a minimum $p < 0.05$; different letters indicate significant difference ($p < 0.05$) compared to control animals (not shown as uninfected animals do not present worms in mesentery).

Figure S2

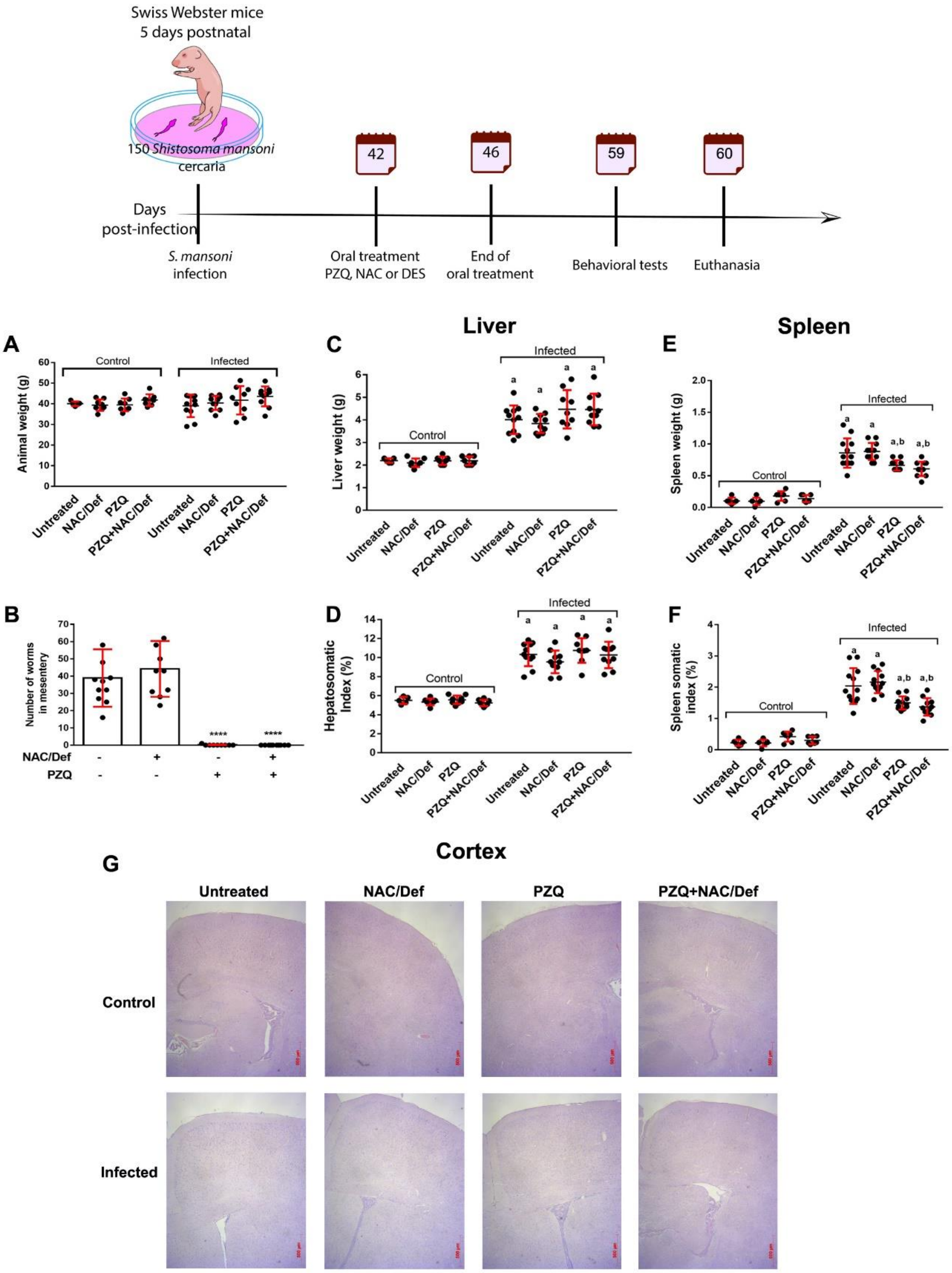

Figure S2. Effect of anthelmintic and antioxidant treatments over somatic parameters of disease and confirmation of parasite absence in the brain of mice infected with *Schistosoma mansoni*. To evaluate the effect of anthelmintic and antioxidant treatments, infected animals were treated with the anthelmintic praziquantel (PZQ) and the antioxidants N-acetyl-L-cysteine (NAC) and deferoxamine (Def). The compounds were administrated daily, by gavage, from the 42nd to the 46th day after infection, and euthanasia was performed at the 60th day post infection. NAC and Def were administered at 200 mg/Kg each, while PZQ was administered at 100 mg/Kg. Somatic and parasitological parameters were evaluated: **A)** animal weight, **B)** number of worms recovered in mesentery, **C)** liver weight, **D)** spleen weight, **E)** hepatosomatic index (liver weight/animal weight*100), **F)** spleen-somatic index (spleen weight/animal weight*100). **G)** Histologic images of prefrontal cortex (representative images from two different experiments; scale bars correspond to 500 µm). Groups were compared with two-way ANOVA with Tukey's post hoc test. Asterisks denote significance degrees for a minimum $p < 0.05$; *a*=different from respective uninfected control; *b*=different from infected, untreated group.

Figure S3

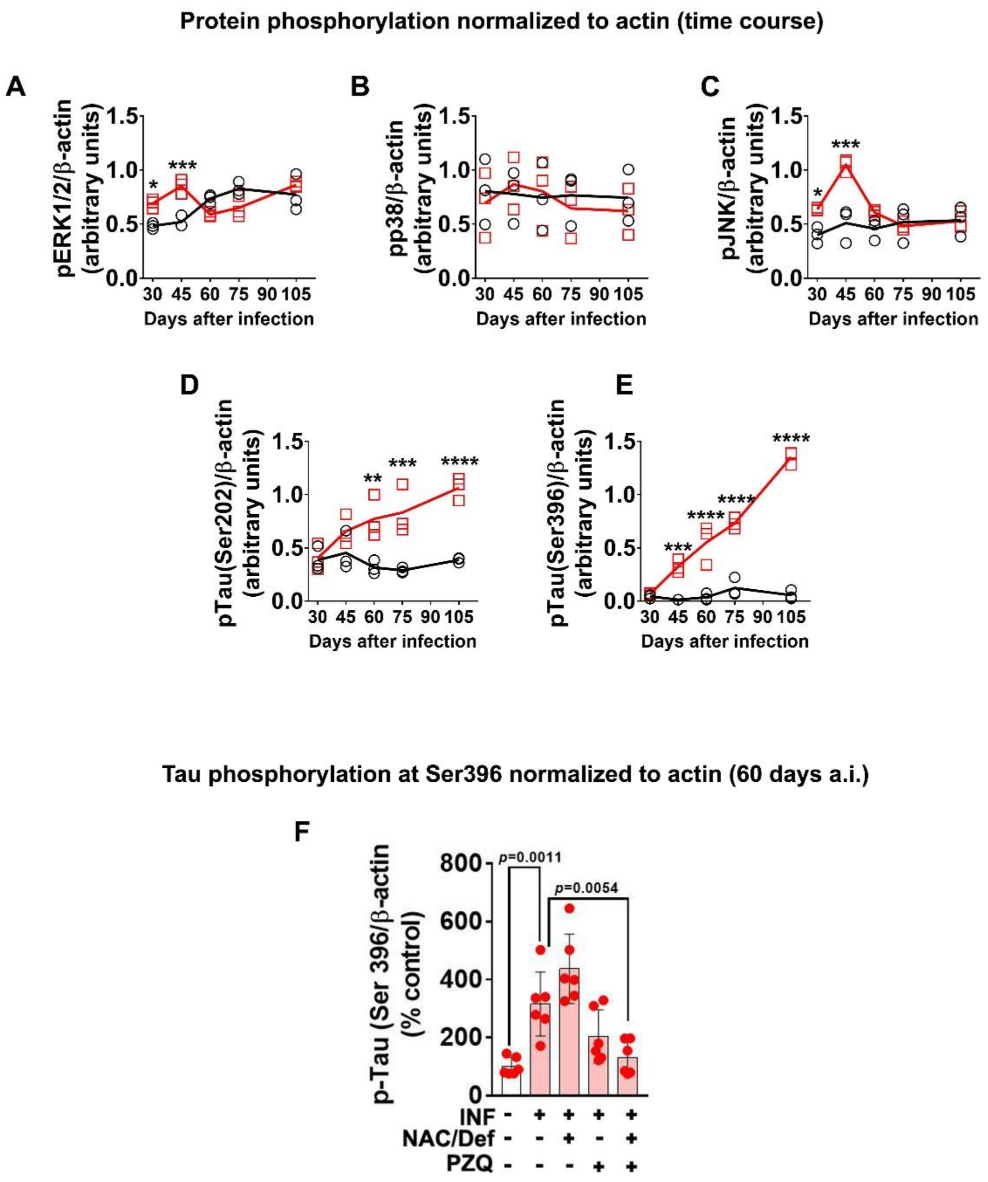

Figure S3. Protein phosphorylation normalized to β-actin content. Five-days-old mice were infected by 150 ± 10 cercariae and euthanized at 30, 45, 60, 75 and 105 days after infection (see *Fig. S1* for experimental design) to isolate prefrontal cortex tissue for WB. Analysis of the phosphorylated/total isoforms of the MAPKs **A)** ERK1/2, **B)** p38 and **C)** JNK, and of the protein Tau phosphorylated at **D)** Ser202 and **E)** Ser396. Mean values ± SD normalized to β-actin content. Group means were evaluated by multiple *t* tests with correction for multiple comparisons with Holm-Sidak. Each asterisk denotes a significance degree for a minimum of $p < 0.05$. Representative gels are shown at Fig. 2. **F)** Phosphorylated Tau at Ser396from infected mice that received daily PZQ (100 mg/Kg) and/or combination of NAC/Def (200 mg/Kg) from the 42nd to the 46th days after infection and were euthanized at the 60th day after infection. Values represent mean ± SD (n=6). Group means were compared by two-way ANOVA followed by Tukey's post hoc test; p < 0.05 values are embedded in the graphs.

Figure S4

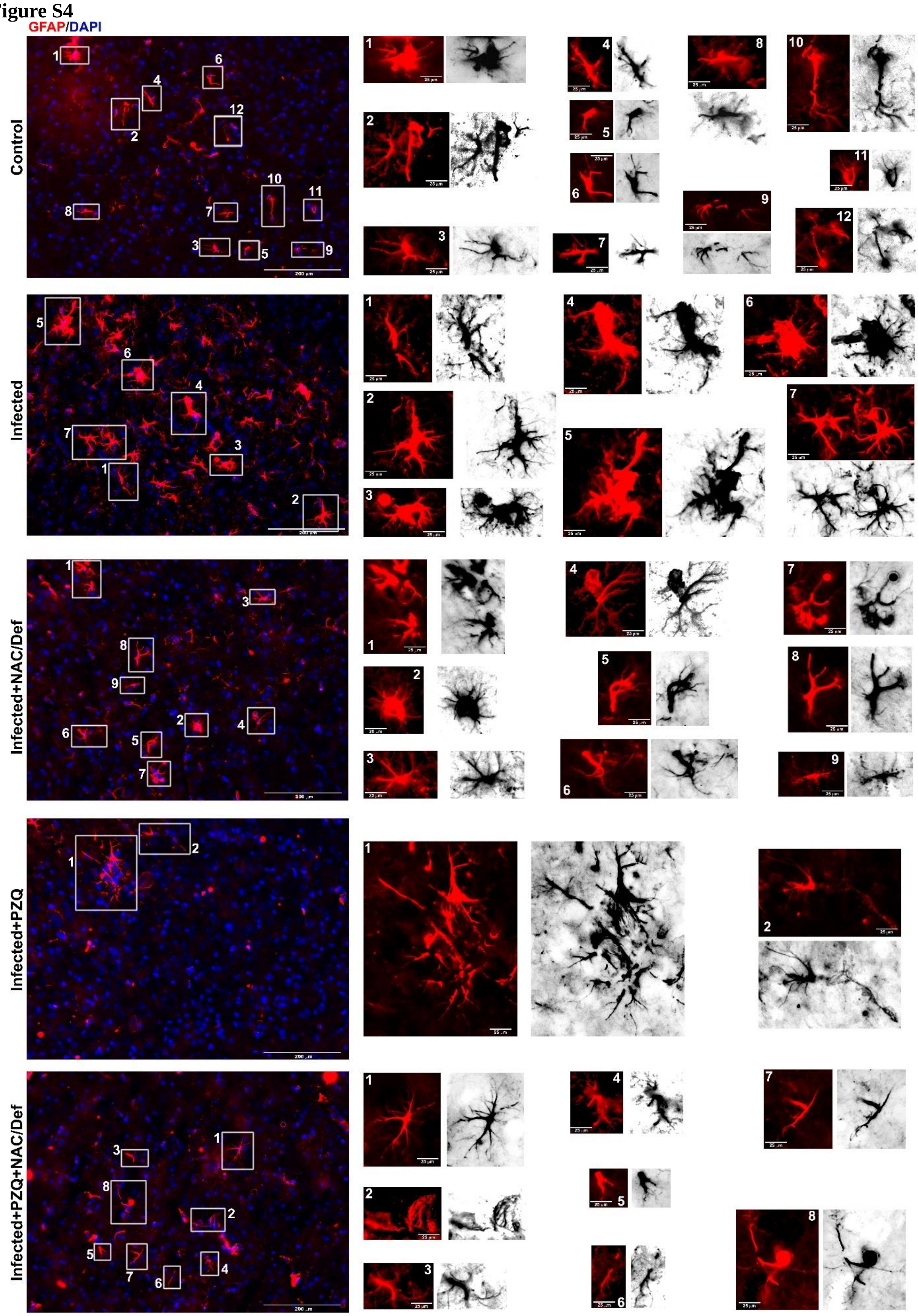

Figure S4: Morphological observation of GFAP immunofluorescence staining in prefrontal cortex of mice infected with *S. mansoni*. Representative sections of tissues from mice from different groups are shown at *left* panels (scale bars length is 200 µm) with DAPI staining. Inserts of isolated cells are detailed at 2.5 x augmented visualization at *right* (scale bars length is 25 µm). Inserts show only red fluorescence (without DAPI) and its grayscale reproduction for optimal visualization of cellular processes. Image panels are derived from the same micrographs presented in Figure 4C.

Figure S5

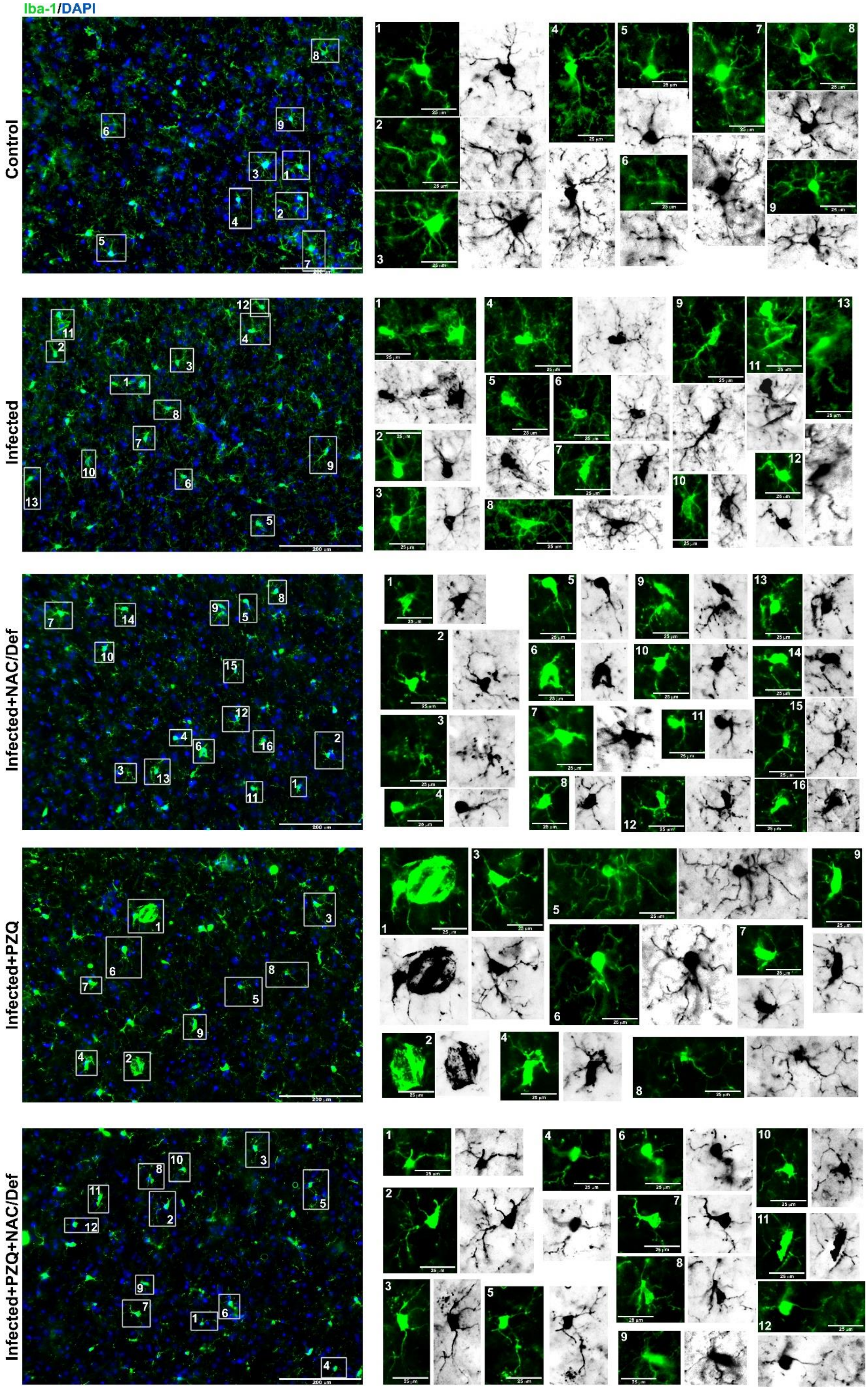

Figure S5. Morphological observation of Iba-1 immunofluorescence staining in prefrontal cortex of mice infected with *S. mansoni*. Representative sections of tissues from mice from different groups are shown at *left* panels

(scale bars length is 200 μm) with DAPI staining. Inserts of isolated cells are detailed at 2.5 x augmented visualization at *right* (scale bars length is 25 μm). Inserts show only green fluorescence (without DAPI) and its grayscale reproduction for optimal visualization of cellular processes. Image panels are derived from the same micrographs presented in Figure 4C.

Figure S6

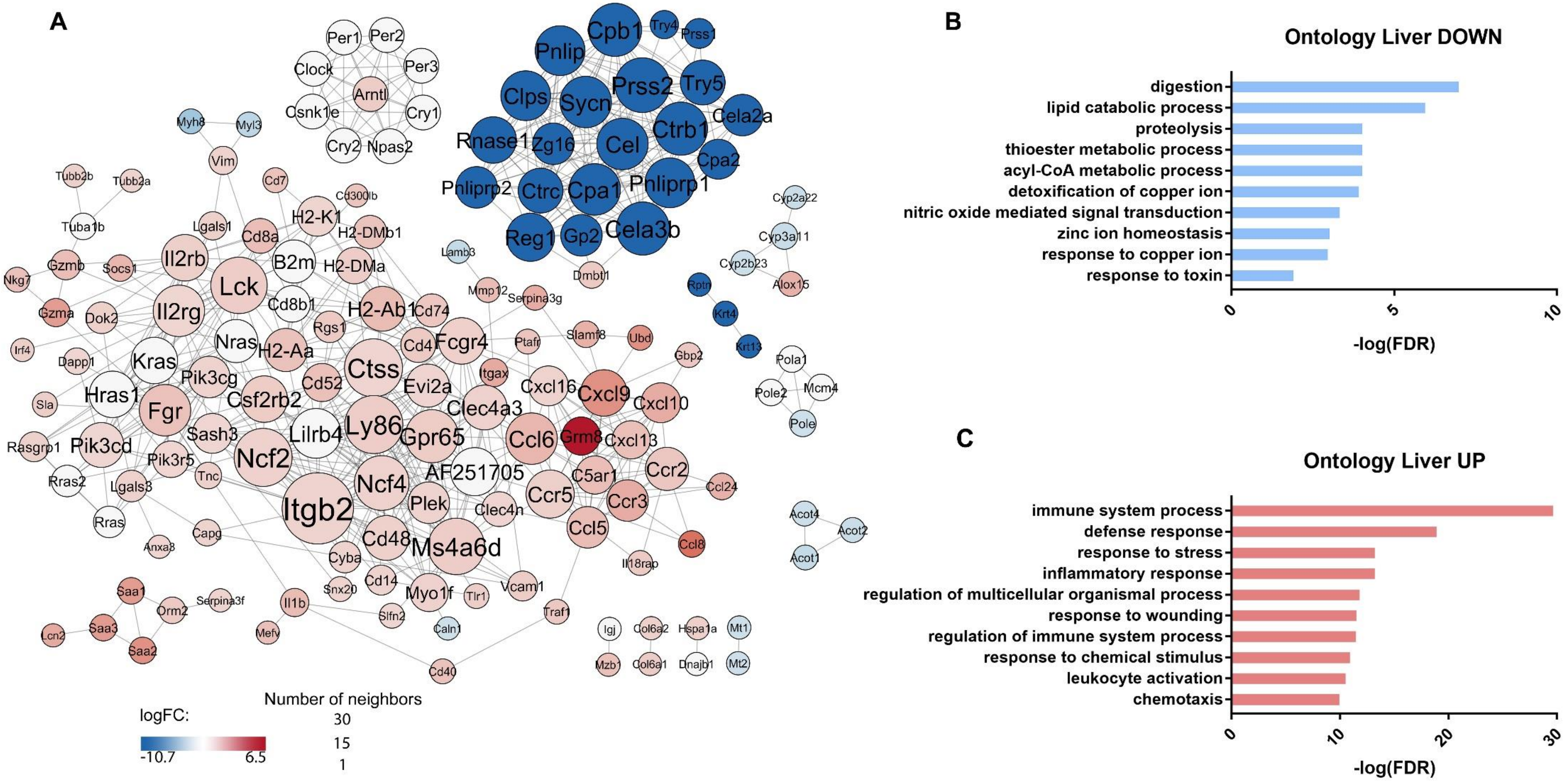

Figure S6. Transcription data collected from mouse liver at 32 days post-infection, RNA-seq data accession is GSE94132 (28650976). Differentially expressed genes (DEGs) were considered to have an FDR < 0.05 and the base 2 logarithmic fold change correction (logFC) major or equal to ±1. Gene expression analysis of RNA-seq data was performed in R with the packages edger (19910308) and limma (25605792). Protein-protein interaction networks (PPINs) were generated using the DEGs as seeds in STRING v.10.5 (25352553). The following parameters were utilized in STRING: all active interaction sources, excluding textmining; 0.400 as minimum required interaction score; no more than 20 neighbors in the first shell; and none for second shell.

Figure S7

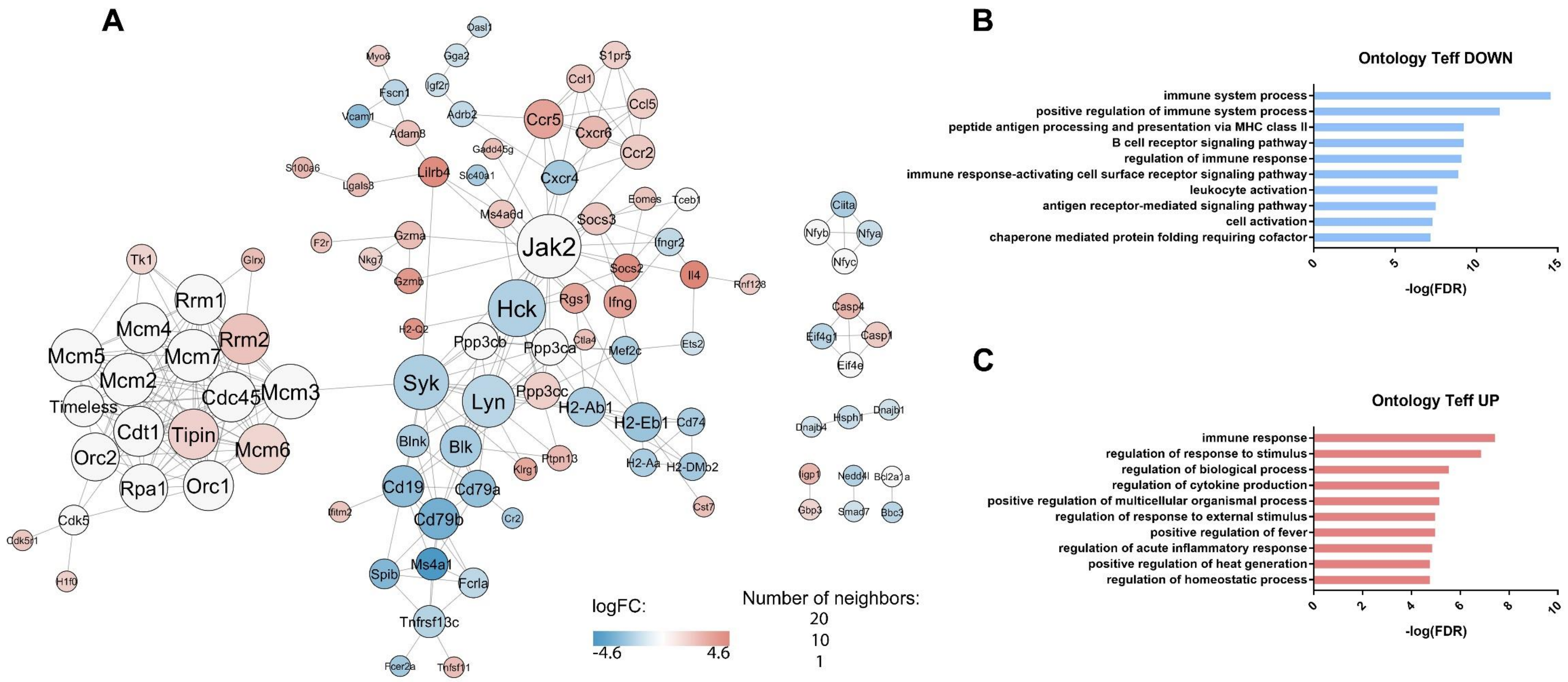

Figure S7. Transcription data collected from T effector (Teff) cells extracted from mouse spleen at 63 days post-infection, microarray data accession GSE17580 (20007528). Differentially expressed genes (DEGs) were considered to have an FDR < 0.05 and the base 2 logarithmic fold change correction (logFC) major or equal to ±1. Microarray data were analyzed with GEO2R tool. Protein-protein interaction networks (PPINs) were generated using the DEGs as seeds in STRING v.10.5 (25352553). The following parameters were utilized in STRING: all active interaction sources, excluding textmining; 0.400 as minimum required interaction score; no more than 20 neighbors in the first shell; and none for second shell.

Figure S8

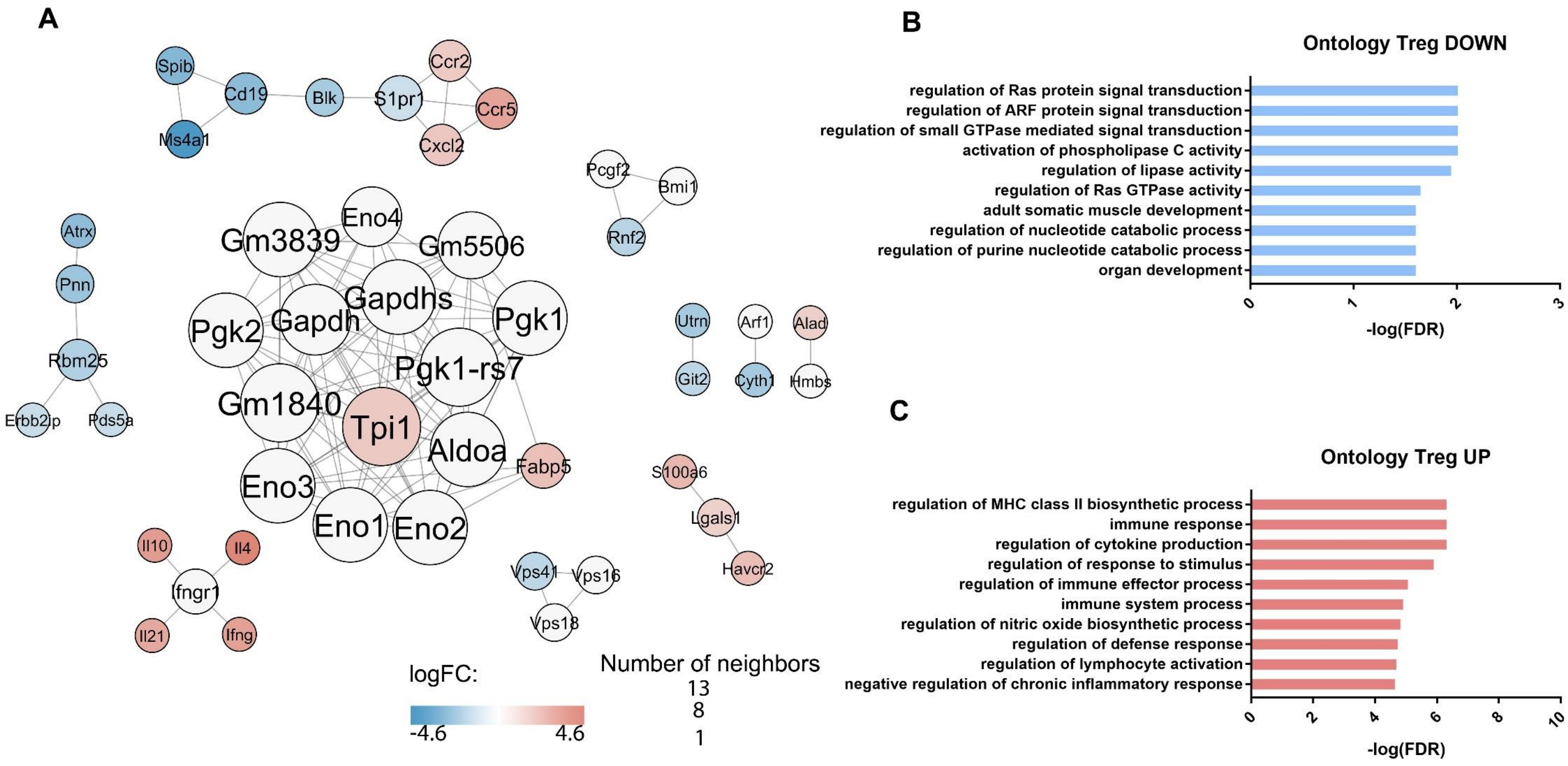

Figure S8. Transcription data collected from T regulatory (Treg) cells extracted from mouse spleen at 63 days post-infection, microarray data accession GSE17580 (20007528). Differentially expressed genes (DEGs) were considered to have an FDR < 0.05 and the base 2 logarithmic fold change correction (logFC) major or equal to ±1. Microarray data were analyzed with GEO2R tool. Protein-protein interaction networks (PPINs) were generated using the DEGs as seeds in STRING v.10.5 (25352553). The following parameters were utilized in STRING: all active interaction sources, excluding textmining; 0.400 as minimum required interaction score; no more than 20 neighbors in the first shell; and none for second shell.
